# A modelling study of nonlinearity of the cross-shore flow under breaking waves

**DOI:** 10.1038/s41598-026-53784-x

**Published:** 2026-08-03

**Authors:** Gugum Gumbira, Peng Zheng, Ming Li, Yan Zhou, Xiaonan Tang

**Affiliations:** 1 Department of Civil and Environmental Engineering, University of Liverpool, Liverpool, L69 3GQ UK; 2 Research Centre for Hydrodynamics Technology, The National Research and Innovation Agency of Indonesia (BRIN), Yogyakarta, Indonesia; 3 Key Laboratory of Environmental Protection Technology on Water Transport, Ministry of Transport, Tianjin Research Institute for Water Transport Engineering, Tianjing, China; 4 Department of Civil Engineering, Xi’an Jiaotong-Liverpool University, Suzhou, China

**Keywords:** Process-based model, Velocity nonlinearities, Plunging-spilling breaker, Free-stream/near-bed velocity, Wave boundary layer, Ocean sciences, Physics, Solid Earth sciences

## Abstract

A new process-based modelling framework is used to simulate wave-driven flows over a bar in the large-scale SINBAD and LIP11-1C experiments, representing high- and low-wave conditions, respectively. The framework couples an open-source Boussinesq wave model with a one-dimensional vertical (1-DV) column model. Model-data comparisons show good model skill, with discrepancies less than 10*%* for waves and hydrodynamics, providing a robust basis for analysing higher-order velocity moments. The corresponding results demonstrate that the cross-shore evolution of the free-stream velocity skewness and asymmetry depends strongly on the breaker type, especially in the surf zone. In both tests, the skewness and asymmetry increase during shoaling. Under a spilling breaker, the intrawave velocity shapes reach peak nonlinearities near the breaking point and then decay gradually through the surf zone. In the plunging breaker, peak nonlinearities occur around the bar crest, decrease sharply at the bar crest, and recover within the splashing and plunging points. Inside the surf zone, the velocity signals evolve toward a strongly skewed-asymmetrical form. A very consistent pattern is found in the vertical direction across all cross-shore regions. The velocity skewness systematically increases towards the bed, while the asymmetry decreases, resulting in a more skewed, less asymmetric near-bed velocity. Boundary-layer filtering, phase shift, and undertow contribute to the velocity transformation inside the wave boundary layer. The bispectral analysis shows that, despite spectral energy decay with depth, strong triad coupling and a coherent biphase pattern persist, and at some locations, intensify within the wave boundary layer, demonstrating breaking influences on the re-organised harmonic interactions rather than making the flows random. These associated findings imply that sediment transport formulations based on free-stream skewness and asymmetry may misrepresent near-bed sediment transport.

## Introduction

Accurately modelling onshore sediment transport remains challenging, despite its central role in post-storm beach recovery and sandbar migration^1^. This process governs the episodic shoreline advance and retreat observed in nature and underpins predictive models of coastal change. The debate over the long-term survivability of sandy beaches under sea-level rise^2,3^ further highlights the need to resolve the physical mechanisms controlling wave nonlinearity^4^, which exerts a strong influence on coastal morphological response. Recent studies have increasingly highlighted wave nonlinearity, that is commonly quantified through velocity skewness and asymmetry (acceleration skewness), as a key driver of onshore-directed sediment transport^5–8^. However, understanding remains limited regarding how these nonlinearities vary spatially across the beach profile, particularly under different wave-breaking regimes. Improved quantification of these processes is essential for advancing cross-shore sediment transport models capable of capturing both storm-driven erosion and subsequent morphological recovery^9^.

The wave velocity skewness (*Sk*) is a measure of the statistical bias of the horizontal orbital velocity, while the asymmetry (*As*) quantifies the steepness of the front and back waveforms. Their magnitudes can be computed using following Eq. (1) for skewness and Eq. (2) for asymmetry respectively:

1 $$\begin{aligned} Sk= & \frac{\overline{(\psi - \bar{\psi })^3}}{\psi ^3_{rms}} \end{aligned}$$

2 $$\begin{aligned} As= & -\frac{\overline{\Im H(\psi - \bar{\psi })^3}}{\psi ^3_{rms}} \end{aligned}$$

where *ψ* is a physical parameter, such as flow velocity in the present study. The over bar “$\bar{~}$” defines a averaging procedure, and *ℑ H* represents Hilbert transform function. It is shown that these equations link the cubic values of the signals in the time domain with the third-order moment in the frequency domain. The skewness is tied to the real part of the bispectrum, whereas the asymmetry is connected with the imaginary part^10^. Based on Eq. 1 and 2, an unmodulated sinusoidal wave has *Sk = 0.0* and *As=0.0*, reflecting a perfectly symmetrical wave crest and trough with front and back faces that are mirrored.

Across the beach profile, the distribution of wave velocity skewness and asymmetry at the free-stream level is primarily governed by the evolving surface wave shape as offshore waves propagate shoreward, transforming from nearly sinusoidal to increasingly nonlinear forms through interactions with the seabed during shoaling and subsequent breaking. In the shoaling zone, decreasing water depth causes wave crests to sharpen and troughs to broaden, producing a skewed waveform. Bispectral analyses of field observations^10^ demonstrated that the nonlinear energy transfer from fundamental to higher harmonics during shoaling generates skewed and asymmetric surface elevations. The resulting orbital velocities exhibit higher magnitudes and shorter durations under the wave crest, enhancing both skewness and asymmetry. As waves break, the original waveform transforms into a forward-leaning, sawtooth-like shape with a steep front face and gentle rear slope, leading to increased velocity accelerations but reduced skewness^11^. Breaking bores and short waves further modify near-surface orbital velocities.

Attempts have been made in the early stage to quantify local wave skewness and asymmetry based on a nonlinear index^12^ in order to reconstruct the corresponding velocity time series. To incorporate the effects of random waves, the nonlinearity index was linked with wave phase (*ϕ*) to wave height, period, and depth^7^. More recently, emphasis was put on the importance of non-local parameters, such as beach slope, in controlling velocity skewness and asymmetry across the surf zone^11^.

From a sediment transport perspective, near-bed velocity skewness and asymmetry are far more critical to sediment entrainment and transport than that at the free-stream level. However, the near-bed dynamics are affected not only by the evolution of wave shape, but also by the wave boundary layer (WBL) process. It has been shown that the WBL selectively damps high-frequency oscillations and induces phase shifts between the free-stream orbital velocity and near-bed velocity, thereby moderating skewness and asymmetry at the bed surface^13^. A constant phase shift of approximately $25^o$ near the bed was found with an amplification factor for the velocity magnitude of 0.2^14^. Oscillatory tunnel experiments^15^ demonstrated that phase leading at the bed surface can enhance near-bed velocity skewness relative to the free stream. Experimental observations^16^ further revealed significant transformations of both parameters within the WBL, confirming that boundary layer processes strongly modify near-bed flow structure. Direct numerical simulations^17^ examined acceleration-skewed oscillatory flow over a smooth bed and showed that turbulence generation during the accelerating and decelerating phases contributes to increased near-bed skewness. More recently, velocity skewness and asymmetry under modulated wave groups^18^ have been examined, highlighting the varying influence of the WBL on different combinations of skewed and asymmetric wave conditions.

Most of the existing studies have focused on specific cross-shore locations under shoaling or non-breaking or near-breaking waves. Under breaking waves, the near-bed boundary layer is highly distorted, with enhanced turbulence, flow separation, and possibly reversed flows (undertow), which complicates the direct correlation between the free stream skewness and asymmetry with that at the bed surface. Moreover, the influence of different types of breakers, such as plunging breakers during storms and spilling breakers under mild conditions, on near-bed velocity nonlinearity remains poorly understood. Recent experimental evidence suggested the onshore transport due to near-bed wave nonlinearity even under a highly energetic plunging breaker^19^.

This limited understanding of how velocity skewness and asymmetry evolve vertically and across the beach profile introduces substantial uncertainty in sediment transport predictions that rely on free-stream orbital velocities. To address this gap, the present study aims to investigate how wave propagation and breaking transform velocity skewness and asymmetry within the wave boundary layer. Achieving these objectives requires accurate characterisation of wave nonlinearity across the beach. We therefore employ the weakly dispersive Boussinesq model Funwave^20^ to simulate wave propagation and capture surface-level wave nonlinearity, while recognising that its depth-integrated formulation does not resolve vertical flow structure. To complement this limitation, we develop a phase-resolving one-dimensional vertical (1DV) model that estimates near-bed velocity nonlinearity and resolves instantaneous velocity and turbulence characteristics inside and outside the wave boundary layer. The coupled Funwave–1DV framework provides a process-based but computationally efficient approach for representing wave nonlinearity and boundary-layer dynamics across the beach profile. We apply this framework to both plunging and spilling breaking waves, supported by detailed laboratory measurements over a representative bar–trough system. The results reveal how wave breaking and associated energy-transfer processes shape the spatial distribution of wave nonlinearity across the beach, thereby improving the physical basis for cross-shore sediment transport modelling.

## Numerical model

### Wave model

A phase-resolving Boussinesq wave model, FUNWAVE (version 3.5), is employed to simulate wave propagation and wave-induced currents across the shoaling and surf zones^20–22^. Funwave solves the fully nonlinear, weakly dispersive, depth-integrated Boussinesq equations with non-hydrostatic pressure. Wave breaking and run-up are represented through an eddy-viscosity dissipation scheme^22^. The model incorporates wetting–drying, absorbing–generating wave makers for monochromatic and irregular waves, and sponge layers at open boundaries. Simulations are conducted on a Cartesian grid using a hybrid finite-difference/finite-volume discretisation. This configuration has been extensively validated for nearshore wave transformation, breaking, run-up, and wave–current interaction^20–22^. The depth-integrated governing equations are defined as follows:,

3 $$\begin{aligned} & \frac{\partial \eta }{\partial t} + \nabla \!\cdot \!{\textbf{M}} = 0, \end{aligned}$$

4 $$\begin{aligned} & \frac{\partial {\textbf{u}}}{\partial t} + {\textbf{u}}\!\cdot \!\nabla {\textbf{u}} + g\,\nabla \eta + \mathcal {D}(\eta ,{\textbf{u}}) = {\textbf{S}}_{\textrm{diss}}, \end{aligned}$$

where *η (x,y,t)* is the free-surface elevation, *H=h+η* is the total water depth, *h* is the depth below the mean water level, ${\textbf{M}}$ is the horizontal volume flux, ${\textbf{u}}$ is a velocity at a representative depth, $\mathcal {D}(\cdot )$ collects the Boussinesq dispersive terms, and ${\textbf{S}}_{\textrm{diss}}$ includes bottom friction and breaking-induced turbulence (eddy viscosity/roller or TVD dissipation). ${\textbf{M}}=H\,\bar{{\textbf{u}}} + \mathcal {O}(\nabla \nabla {\textbf{u}})$, with explicit expressions for the dispersive operators given in Funwave^22^.

Wave breaking is a critical process that must be represented with sufficient accuracy to resolve the associated hydrodynamics. In this study, energy dissipation during breaking is modelled using an eddy-viscosity formulation^22^. However, because Boussinesq-type models cannot fully capture the onset of overturning or the subsequent dissipation of kinetic energy–particularly for energetic plunging breakers^23^, the choice of breaking parameterisation is essential. To better represent the distinct dynamics of spilling and plunging waves, different breaking initiation and termination schemes are implemented in the present simulations^24,25^. For a spilling breaker, the surface variation criterion is used, e.g. a breaking event begins when $\frac{\partial \eta }{\partial t} > C_{brk1}\sqrt{gH}$, and the wave will continue to break until $\frac{\partial \eta }{\partial t} < C_{brk2}\sqrt{gH}$. These parameters $C_{brk1}$ and $C_{brk2}$ are recommended with a value of 0.45 and 0.35, respectively. For large energetic wave breaking, such as a plunging breaker, the local slope angle criterion is used, e.g. $\frac{\partial \eta }{\partial x}$ exceeds an incident breaking angle ($\alpha _b$), taking into account the local bed slope $\frac{\partial h}{\partial x}$:

5 $$\begin{aligned} \frac{\partial \eta }{\partial x}- \frac{\partial h}{\partial x}> \tan (\alpha _b) \end{aligned}$$

The jet-induced bore in the plunging breaking waves tends to roll a longer distance across the surface than that under a spilling breaker. To represent this difference, it is assumed that the plunging breaking process continues until the water level drops below zero, and the breaking position moves to another location as detected by Eq. (5).

Under plunging breaking waves, the free-stream orbital velocity is governed primarily by the oscillatory motion beneath the overturning crest rather than by the details of the overturning itself. The wave boundary layer further damps high-frequency disturbances as they propagate downward, limiting the influence of the breaking jet on near-bed velocity. Consequently, even though the Boussinesq model does not explicitly resolve the geometric overturning of plunging waves, it can still reproduce the essential velocity nonlinearity at both free-stream and near-bed levels. This is confirmed by the close agreement between predicted and measured velocities, skewness, and asymmetry presented later. Moreover, the bispectral analysis confirms that near-bed energy distributions remain relatively insensitive to the breakdown of the free surface and are instead controlled by the underlying oscillatory motion and boundary-layer dynamics.

In this study, the alongshore dimension is assumed to be uniform, and periodic boundary conditions are applied laterally. In the cross-shore direction, Funwave employs offshore and onshore boundary conditions. At the offshore boundary, a standard generating–absorbing condition with a sponge (relaxation) zone is used, where the target incident wave field, monochromatic or irregular, is imposed within the inner sponge region to force wave-induced motions in the domain. Free-surface elevation and depth-averaged velocities are smoothly relaxed using spatially varying damping to minimise wave reflection. At the shoreward boundary, a wetting–drying scheme enables dynamic run-up and run-down on the beach face, with a prescribed critical water depth defining the moving shoreline. A zero normal velocity condition is applied near the landward limit. Sponge layers are also placed shoreward of the wave generator and seaward of the shoreline boundary to attenuate reflected waves. The bed friction is typically represented by a uniform friction factor of 2.5 mm for the plunging test and 1.5 mm for the spilling test.

### 1-DV point model

To resolve the vertical structure of horizontal velocity and turbulence, a local one-dimensional vertical (1-DV) model is embedded at each computational node of the Funwave domain. Within the 1-DV framework, the pressure field is decomposed into an oscillatory component associated with wave-induced motions and a mean component arising from the local wave-averaged surface slope. The vertical extent of the model reaches from the seabed to the free surface, or to the level immediately below the wave roller in breaking regions, allowing consistent representation of both nonbreaking and breaking wave conditions. The horizontal momentum equation is formulated as^26^:

6 $$\begin{aligned} \frac{\partial u^x}{\partial t}=-\frac{1}{\rho _w}\frac{\partial p}{\partial x}+\frac{\partial }{\partial z}\left( \left( \nu _t + \mu \right) \frac{\partial u^x}{\partial z} \right) \end{aligned}$$

where $u^x$ is horizontal velocity, *p* is the fluid pressure, $\rho _w$ is fluid density, *t* is time, *z* is the vertical coordinate, $\nu _t, \mu$ are the turbulence eddy viscosity and kinematic viscosity, respectively. The pressure gradient is computed as:

7 $$\begin{aligned} -\frac{1}{\rho _w}\frac{\partial p}{\partial x}=\frac{\partial u_f}{\partial t}-g\frac{\partial \bar{\eta } }{\partial x} \end{aligned}$$

where $u_f$ and $\bar{\eta }$ are wave-induced orbital velocity and mean free surface elevation calculated by FUNWAVE, respectively. *g* is the gravitational acceleration. The first term on the right-hand side of the equation represents the wave-induced oscillatory motions along the direction of wave propagation, while the second term is the setup-induced steady current in the offshore direction (undertow).

A *k*-*ε* turbulence model^27^ is used in the present study to calculate turbulence kinetic energy *k*, dissipation rate *ε* and eddy viscosity $\nu _t$:

8 $$\begin{aligned} \frac{\partial k}{\partial t} = PROD + \frac{\partial }{\partial z}\left( \frac{\nu _t}{\sigma _k} \frac{\partial k}{\partial z} \right) - \epsilon \end{aligned}$$

9 $$\begin{aligned} \frac{\partial \epsilon }{\partial t}=\frac{\partial }{\partial z}\left( \frac{\nu _t}{\sigma _{\epsilon }} \frac{\partial \epsilon }{\partial z} \right) +C_{\epsilon 1}\frac{\epsilon }{k}PROD-C_{\epsilon 2}\frac{\epsilon }{k} \end{aligned}$$

The turbulence eddy viscosity is given as:

10 $$\begin{aligned} \nu _t=C_{\mu }\frac{k^2}{\epsilon } \end{aligned}$$

where *k* and *ε* are the turbulent kinetic energy and dissipation rate, respectively. $C_{\epsilon 1}$, $C_{\epsilon 2}$, $\sigma _{k}$, and $\sigma _{\epsilon }$ are the model constants with values of 1.44, 1.92, 1.00, and 1.30, respectively. $C_{\mu }$ is taken as constant with a value of 0.09. The turbulence kinetic energy production term includes both turbulence stress due to the wave motion and the breaking process:

11 $$\begin{aligned} PROD=\left( \frac{\partial u^x}{\partial z} \right) ^2 + Prod_{brk} \end{aligned}$$

Extra turbulence kinetic energy $Prod_{brk}$ is injected into the top boundary during the passing of the roller at the water surface^26^.

At the bed (*z=0*), the 1-DV model applies a non-slip boundary condition for the velocities. The turbulence model applies a local equilibrium condition:

12 $$\begin{aligned} k=\frac{u_s^2}{\sqrt{c_1}} \epsilon =\frac{u_s^3}{\kappa z(0)} \end{aligned}$$

where $u_s$ is the friction velocity near the bed, calculated using a logarithmic profile; $c_1$ = 0.09 is a coefficient, and *z*(0) is the elevation where velocity becomes zero. The near-bed roughness height is assigned according to $3d_{50}$ where $d_{50}$ is the local median grain size. At the water surface, both velocity and turbulent equations adopted zero-gradient boundary conditions. However, when breaking takes place, the velocity at the surface boundary is given as the corresponding roller velocity ($u_{rol}$):

13 $$\begin{aligned} u^x(z=H) = u_{rol} = \alpha \sqrt{gH} \end{aligned}$$

where $\alpha \in [0.1,1.3]$ is the model calibration parameter, following the concept of Schäffer^28^. In their approach, surface roller model was introduced to include the momentum deficit of breaking waves. The roller is assumed to travel at an equal speed with the shallow water celerity, and the resulting energy dissipation depends on the roller volume.^29^ calibrated the *α* value against experiment and found that *α =* 1.3 provide accurate wave height decay across the surf zone. In the present study, a series of tests was conducted with different values of *α*, and *α =* 1.3 yielded the best model performance.

As mentioned above, the roller is also assumed to ride on top of the boundary when passing the local point during the period from the wave crest to the crest-trough crossing. Once the roller propagates over the local point, the velocity at the top boundary resumes the zero-gradient condition.

It is important to note that the prescribed roller velocity is applied at the top of the surface boundary, which lies just outside the computational domain of the 1DV model. As a result, its influence is limited to the uppermost portion of the simulated velocity profile. The subsurface motion remains primarily governed by the wave-induced oscillatory pressure gradient and the mean water-level slope (setup), such that the depth-averaged velocity produced by the 1DV model is consistent with that computed by FUNWAVE. The close agreement between the predicted mean flow and the FUNWAVE solution further demonstrates the dynamical compatibility between the 1DV formulation and the Boussinesq framework.

## Numerical simulations

The model is applied to two contrasting hydrodynamic conditions. The first case (SINBAD) represents a storm-like, wave-driven flow with a plunging breaker^19^, whereas the second case (LIP11-1C) corresponds to a less energetic, spilling-breaker regime^30^. In both experiments, a shore-parallel sandbar is present adjacent to the offshore-facing slope. Comprehensive measurements of hydrodynamic and sediment parameters span the shoaling, breaking, and surf-zone regions, providing a robust dataset for model evaluation.

**Fig. 1:**
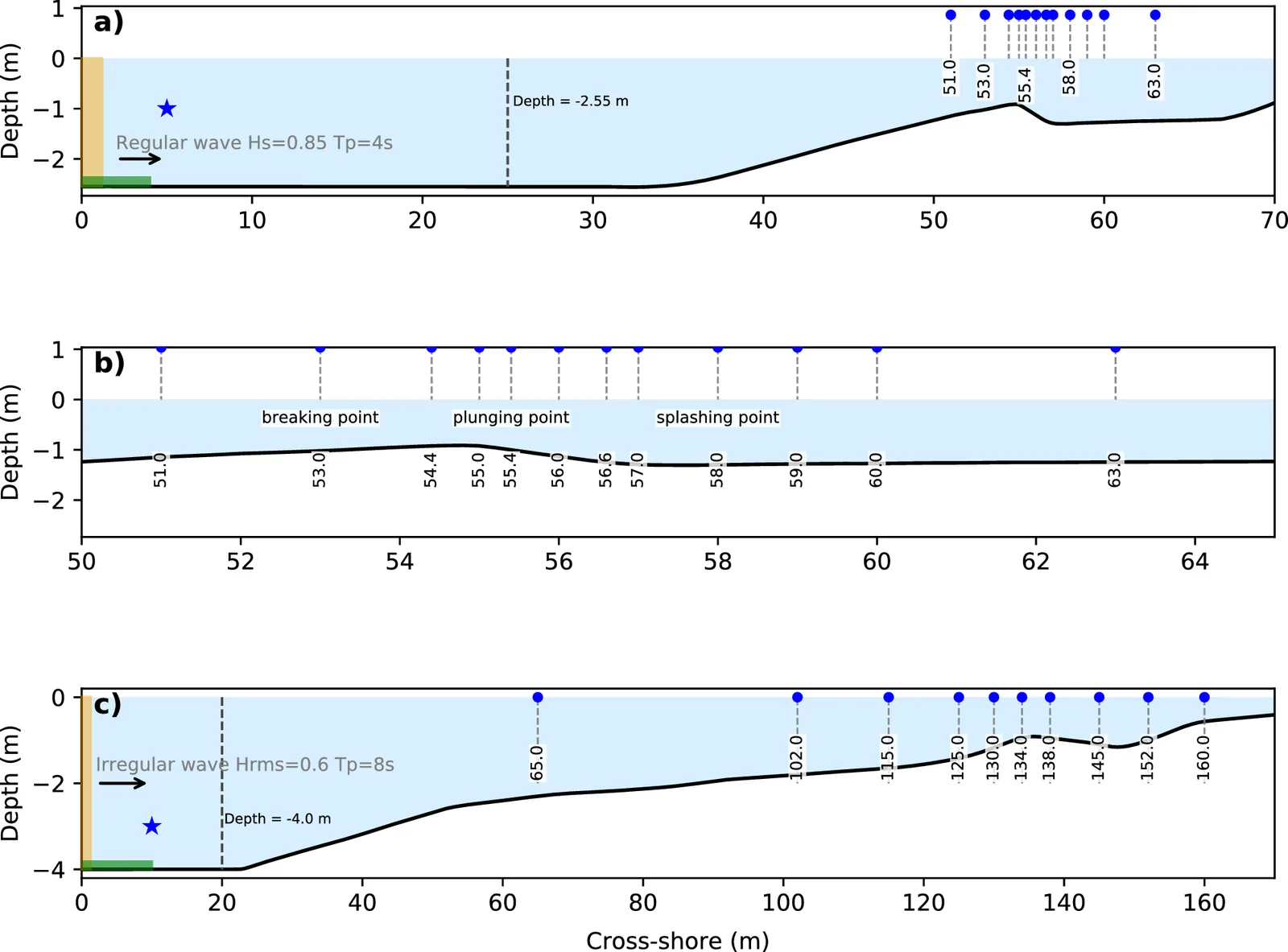
Experimental and numerical setup for the SINBAD test (**a**), detail representation around the bar (**b**) where breaking, splashing, and plunging points are defined, and (**c**) domain study of the LIPII-1C tests. Circles represent measurement positions for each case, while stars are internal wave makers in the numerical domain. The green blocks present sponge layers to dump reflected waves from the offshore side.

### SINBAD case

A large-scale SINBAD experiment^19^ has been conducted to study the effects of plunging breakers on near-bed hydrodynamics and sediment transport. The experiment was carried out at the Universitat Politècnica de Catalunya (UPC) using a wave flume measuring 100 m long, 3 m wide, and 4.5 m deep. The bed profile consisted of a 1:10 offshore-facing slope, followed by a sandbar formation, a 1:7 shoreward-facing slope, a 12 m dissipative/flat beach part, and a 1:7.5 sloping beach covered by geotextile and concrete. Medium sands (median diameters $D_{50} = 0.24$ mm) was placed from *x = 35* to *x = 68* m. At the wave paddle, where the still water depth was 2.55 m, regular monochromatic waves with a height of 0.85 m and a period of 4 s were generated. The breaking index (surf similarity = 0.56) indicates the plunging breaker type. Figure 1a shows the setup of the instruments involved in the experiment^19^. Three regions^31^ across the test section are defined as the breaking (x = 53 m), plunging (x = 55.5 m), and splashing points (x = 58.5 m), as shown in Fig. 1b.

The model was set up with a domain of 3 m wide, 200 m long, and 2.55 m deep, discretised with a uniform 0.2 m horizontal grid and 35 non-equidistant levels in the vertical direction (grid sizes varying from 0.001 to 0.05 m). The wave forcing matched the experimental conditions (*H = 0.85* m, *T = 4* s). Sponge layers of 20 m in length were applied at both offshore and onshore boundaries to minimise wave reflection. The simulation started to simulate wave propagation from the wave-maker to the shoreline using Funwave alone, without coupling with the 1-DV model. Once steady-state conditions were reached, the 1-DV model was initialised, using the same time steps as FUNWAVE to simplify parallelisation. The time step is 0.01 sec, and the simulations were run for a total of 120 minutes. The bed roughness height was set between 0.25 mm and 5 mm according to the experiment. In the SINBAD case the near-bed term covers the region below than 0.005 m, while the free-stream term refers to the level of 0.1 m above the depth, in order to be consistent with the near-bed hydrodynamic measurement of^32^. More detailed parameters can be seen in Table 1.

**Table 1:** SINBAD test model parameters.

Variable	Value	Unit
Offshore water depth	2.55	m
Domain length	200.0	m
Horizontal grid resolution	0.2	m
Time step	0.01	s
Offshore wave height $H_s$	0.85	m
Offshore wave period $T_p$	4.0	s
Vertical grid resolution	0.001–0.05	m
Offshore sponge zone	20.0	m
Onshore sponge zone	20.0	m

### LIP11-1C case

For a comparison with the energetic SINBAD conditions, the LIP11-1C experiment^30^ is used to investigate wave nonlinearity under milder, spilling-breaker conditions. The experiment was conducted in the Deltares wave flume (233 m long, 5 m wide, 7 m deep), where random waves were generated to induce onshore sediment transport and bar migration. At the wave paddle, the water depth was 4.0 m, and the incoming irregular waves followed a narrow-banded JONSWAP spectrum with a significant wave height of 0.6 m and a peak period of 8 s. Measurements were collected at ten cross-shore locations spanning the shoaling, breaking, and surf zones (Fig. 1c). At each station, time-averaged horizontal velocity, turbulent kinetic energy, and suspended sediment concentration were recorded at four vertical levels.

The model domain is 300 m long and 3 m wide, with 4 m of water depth in the flat-bed and wavemaker regions. The horizontal grid resolution is 0.2 m, yielding 1501 grids in the cross-shore direction. Non-uniform vertical grids are used, with 35 *σ* layers of varying resolution from 0.001 to 0.05 m. The near-bed term represent the region less than 0.01 m, while the free-stream level is 0.1 m above the depth. An irregular wave was imposed at the wavemaker section to generate a narrow-banded irregular wave with a 0.6 m incident wave height and an 8 s wave period. Two sponge layers are imposed at the offshore and onshore sides with a 20 m length to minimise wave reflection. More detailed parameters can be seen in Table 2.

**Table 2:** LIP11-1C test model parameters.

Variable	Value	Unit
Offshore water depth	4.0	m
Domain length	300.0	m
Horizontal grid resolution	0.2	m
Time step	0.01	s
Offshore wave height $H_s$	0.6	m
Offshore wave period $T_p$	8.0	s
Vertical grid resolution	0.001–0.05	m
Offshore sponge zone	20.0	m
Onshore sponge zone	20.0	m

### Error statistics

The model’s performance was evaluated using three standard error metrics: the Mean Absolute Error (MAE), Root Mean Square Error (RMSE), and Normalised RMSE (NRMSE). The formulas are given as follows:

14 $$\begin{aligned} {MAE}= & \frac{1}{N} \sum _{i=1}^{N} \left| y_i - \hat{y}_i \right| \end{aligned}$$

15 $$\begin{aligned} \text {RMSE}= & \sqrt{ \frac{1}{n} \sum _{i=1}^{N} ( y_i - \hat{y}_i )^2} \end{aligned}$$

16 $$\begin{aligned} {NRMSE}= & \frac{\textrm{RMSE}}{\bar{y}}, \qquad \bar{y} = \frac{1}{N} \sum _{i=1}^{N} y_i \end{aligned}$$

where $\hat{y}_i$ and $y_i$ are model and experiment values, respectively; *N* is the number of data points.

## Results

### Wave transformation processes

**Fig. 2:**
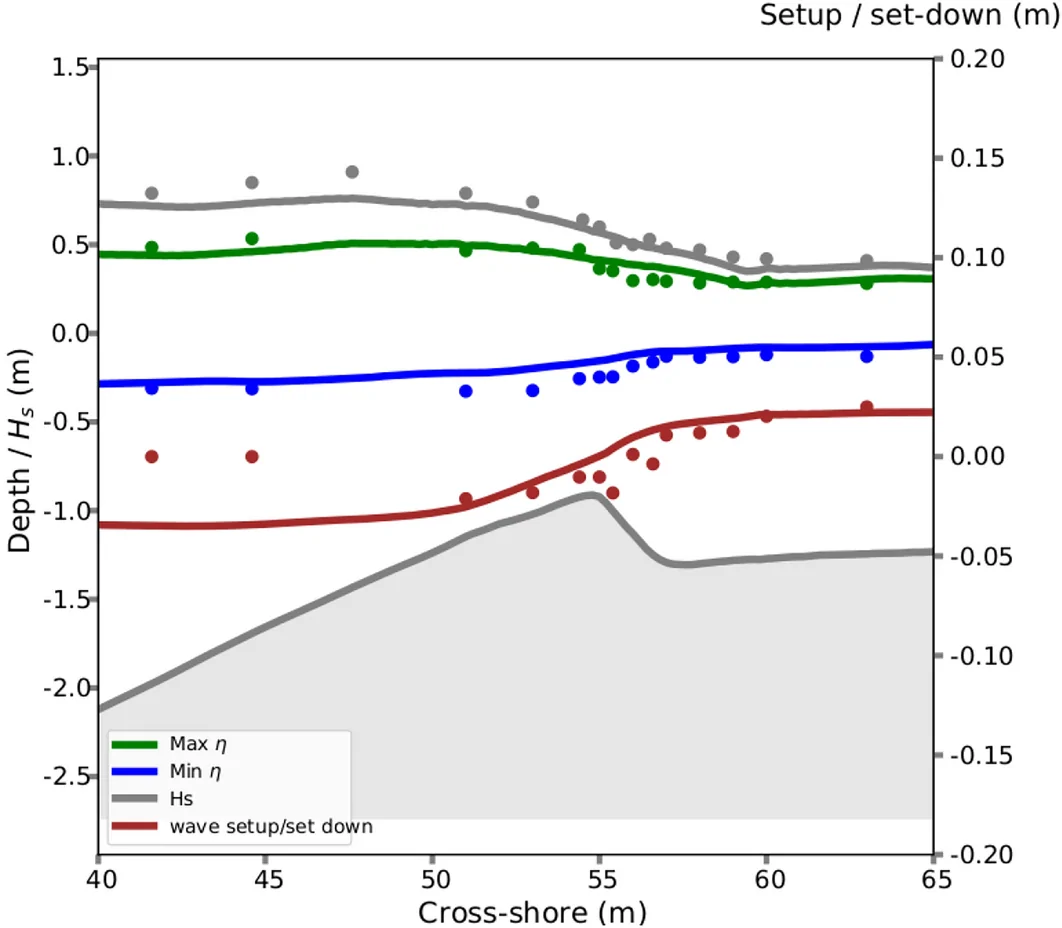
Comparison of computed (lines) wave height (grey), wave crest (green), wave trough (blue), and wave setup/ set down (brown) against experimental data (symbols) in SINBAD test.

Figure 2 compares the simulated and measured wave height, crest–trough envelope, and wave setup for the SINBAD plunging-breaker case. The model reproduces the key stages of wave transformation–shoaling, breaking, and post-breaking dissipation, with good fidelity. As waves shoal over the bar crest, the incident wave height increases from 0.85 to 0.9 m between *x=52.5* m and 53.5 m. Depth-induced breaking initiates near *x=53* m, after which 48–52*%* of the wave energy is dissipated, reducing Hs to approximately 0.4 m by *x=56.6* m. Landward of this point, across the inner surf zone along the plane-bed section (*x=57* – 69 m), the wave height remains nearly constant, indicating stable surf-zone energy conditions.

These variations in wave height are directly reflected in the mean water level response. Shoaling-induced wave growth produces a set-down ($\Delta \eta =- 0.04$ m), whereas post-breaking energy dissipation leads to a set-up ($\Delta \eta =+0.06$ m). Nonlinear energy transfer also reduces wave crest elevations while increasing trough levels after breaking. Overall, the model captures these trends, showing good agreement with measurements^19^. Minor discrepancies occur in the shoaling (*x<50* m) and breaking (*x=55* m) regions, primarily due to strong reflections in the experiment that are not represented in the simulations^33^.

**Fig. 3:**
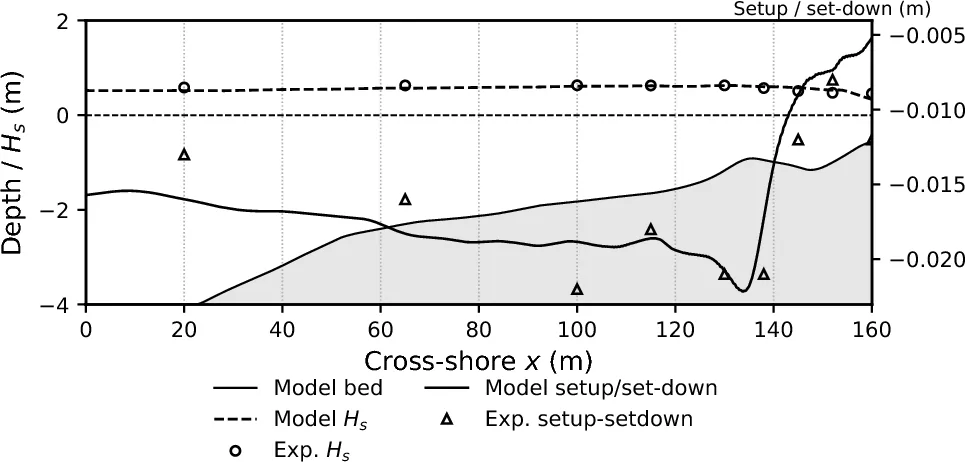
Model-data comparison of significant wave height and wave setup/ set down for LIP11-1C test.

Similarly, Fig. 3 compares the model predictions with measurements of significant wave height and wave setup for the LIP11-1C test. From *x=60* m shoreward, wave heights gradually increase as waves shoal over the sloping beach. Higher waves within the random wave groups break between *80<x<100* m, whereas smaller waves penetrate farther onshore and break between *125<x<140* m. Unlike the SINBAD plunging-breaker case, wave heights in LIP11-1C decrease monotonically once breaking begins, yielding a substantially wider surf zone. Consequently, wave set-down persists from *x=60* m to approximately *x=130* m, after which setup increases rapidly toward *x=150* m. The model reproduces these setup and set-down patterns reasonably well up to *x=150* m. Closer to shore, discrepancies emerge, partly due to the small wave heights and the relatively weak setup compared to the SINBAD case.

### Phase-averaged mean current

**Fig. 4:**
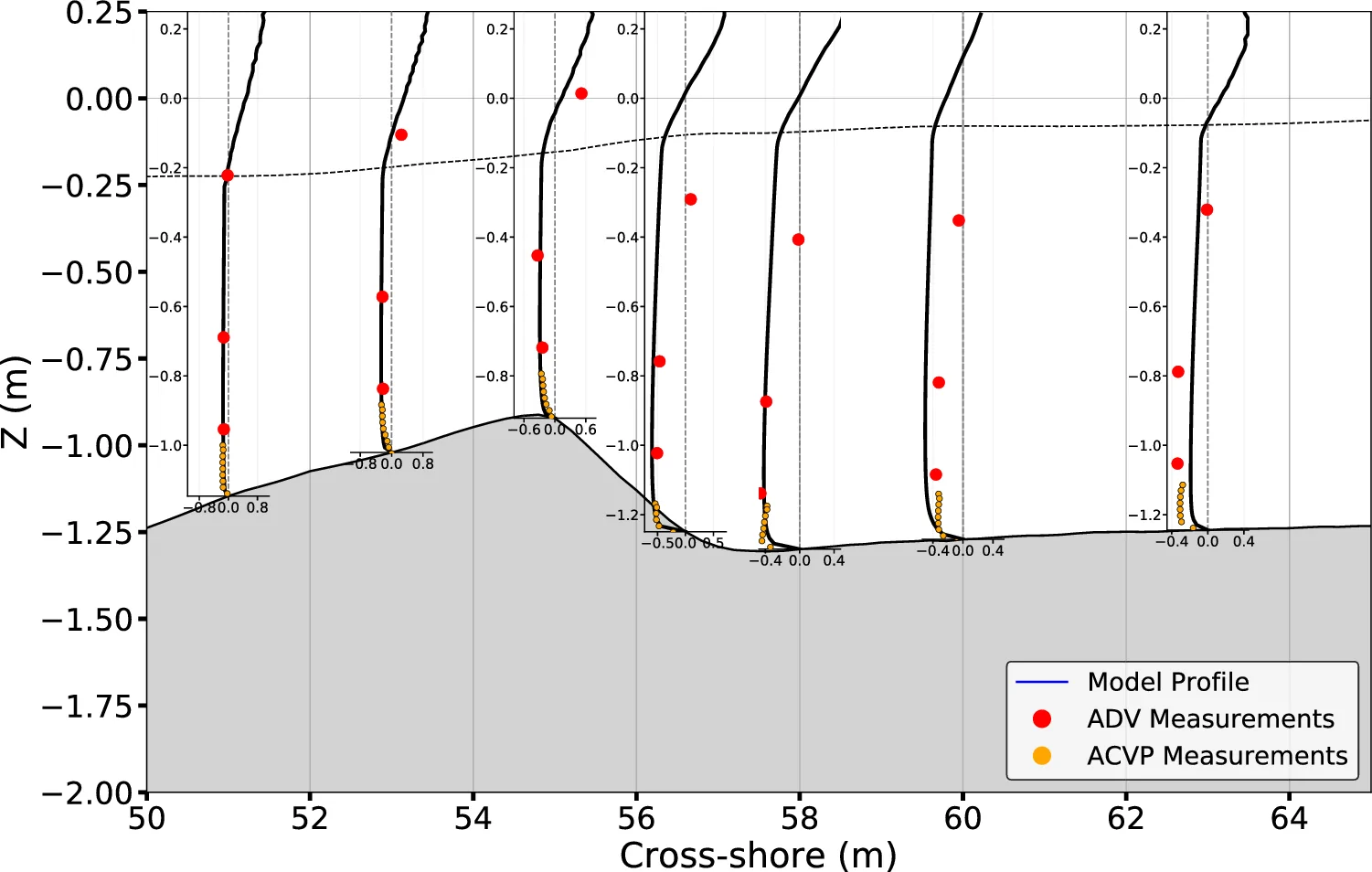
Model-data comparison of time-averaged horizontal velocity profiles (undertow) for SINBAD case. The black dashed lines represent maximum and minimum levels of surface elevation, the blue lines are calculated undertow, red circles are ADV, and yellow circles are ACVP data.

Figure 4 compares the computed phase-averaged mean flow (undertow) with measurements for the SINBAD case. Above the trough level and near the surface, strong onshore-directed flows of 0.5–1.2 m/s develop along the beach. These high-velocity surface currents result from the breaking-wave jet and roller, which inject shoreward momentum into the upper water column as wave crests pass^34^. The onshore flow reaches its maximum near *x=56* m, just shoreward of the breaking point or next to the splashing point, and then gradually weakens to about 0.25 m/s across the outer surf zone. Below the trough level, the flow is dominated by offshore-directed undertow. Prior to breaking (*x=53* m), undertow magnitudes remain weak (*<0.25* m/s) in mid-depth. Shoreward of the breaking point, the undertow strengthens sharply, increasing from about 0.5 to nearly 0.75 m/s toward the bar trough before decreasing slightly in the inner surf zone. Near the bed, both model results and measurements exhibit a logarithmic offshore-directed mean flow, consistent with previous observations^35,36^. The model’s tendency to overpredict near-surface velocities within the surf zone aligns with biases reported in earlier Boussinesq-based studies^33^.

**Fig. 5:**
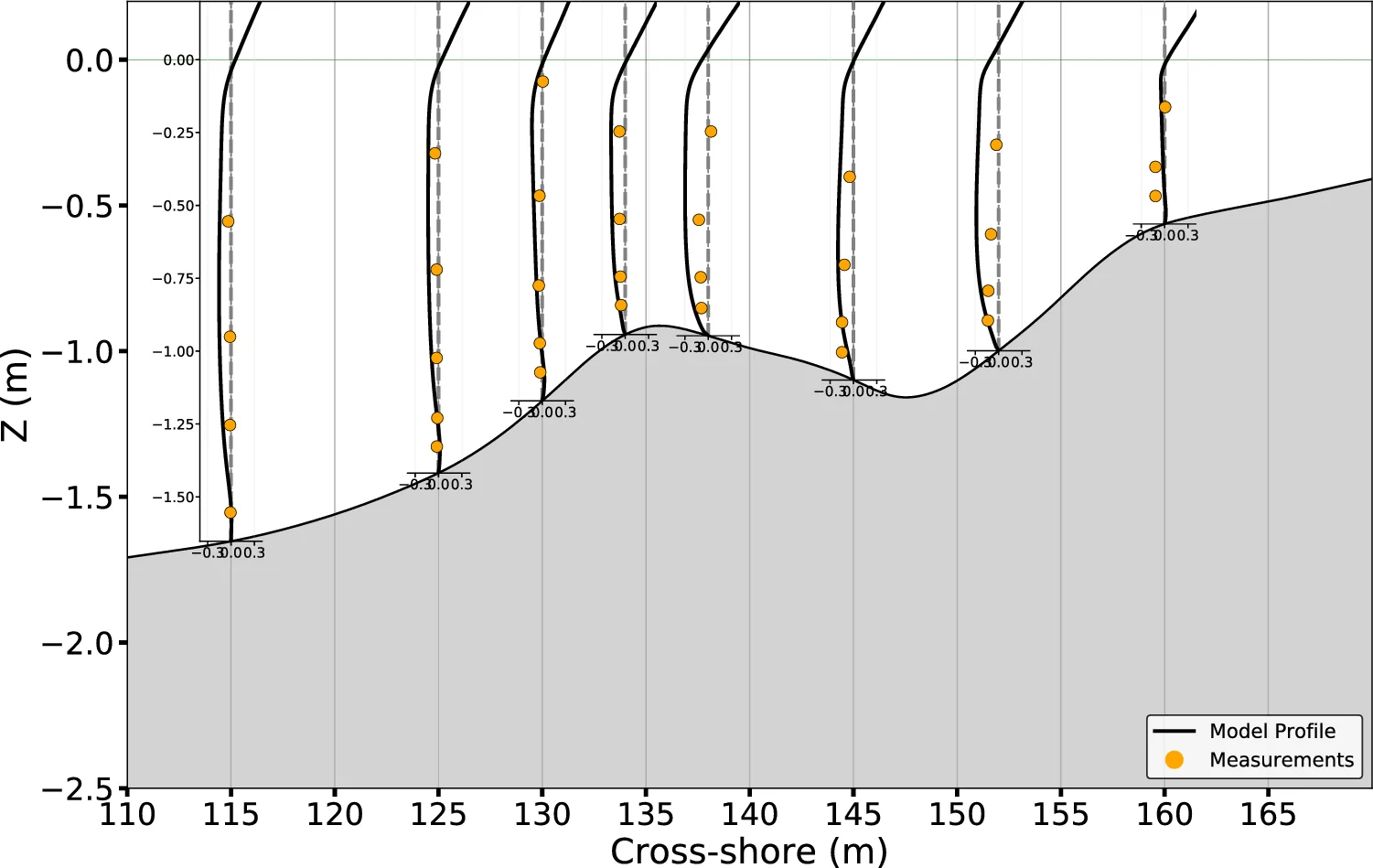
Vertical profiles of time-averaged horizontal velocity (undertow) for LIP11-1C test. Blue lines and symbols are calculated undertow and data, respectively.

Figure 5 compares the predicted undertow profiles for the LIP11-1C test with the measurements. The overall structure resembles that observed in the SINBAD case, although key differences arise from the spilling-breaking regime. In LIP11-1C, breaking energy is distributed more gradually across the surf zone, resulting in weaker roller-driven surface flows. Near the bar crest and trough, frequent breaking events intensify the offshore-directed undertow in the mid–water column, though magnitudes (0.10–0.20 m/s) remain lower than in the plunging-wave conditions of SINBAD. The broadly similar vertical undertow structure across both tests highlights the strong influence of the bar morphology on wave transformation and mean water–level gradients.

Model–data agreement is generally good across the shoaling and breaking regions. Some over-predictions of undertow near the bar crest and trough likely reflect the model’s overestimation of wave setup shoreward of *x>150* m (Fig. 3), which enhances reflected currents relative to the observations. Overall, the model reproduces the characteristic undertow patterns expected over a barred beach.

Table 3 lists the errors for the computed free-surface elevation and velocities against the measurements collected at seven measurement points at the SINBAD test. In the table, *η* is the free surface, $U_{fs}$ is the free stream velocity, $U_{nb}$ is the near bed velocity, $U_{ut,ADV}$ is simulated undertow velocity compared to the ADV data, $U_{ut,ACVP}$ is simulated undertow velocity compared to the ACVP data. The associated metrics are computed from time series data, collected at seven observation points (*x = 51*, 53, 55, 56.6, 58, 60, 63 m), with each station providing 50 wave cycles sample.

The model’s overall skill for resolving free-surface elevation is excellent, with RMSE values ranging from 0.03 to 0.08 m, corresponding to about 7-8*%* of the mean incident wave height ($H_{m0} \approx 1.0$ m). The most significant error is found at *x=53* m where the trough level is under-predicted by 15–20*%*^33^ which is due to the strong reflection close to the bar crest. Excluding the reflection-affected zone reduces RMSE to 0.06 m (6.3*%* of $H_s$) and yields skill scores above 0.92 across 95*%* of the domain. For the near-bed velocity ($U_{\textrm{nb}}$) time series comparison, the highest discrepancy is observed at *x = 53* m (RMSE = 0.28 *m*/*s*) due to the profound effects of the wave breaking. Model-data discrepancy is largest at the plunging region (at *x = 56.6* m), resulting in *RMSE = 0.34 m/s* for the undertow velocity ($U_{\textrm{ut,ADV}}$) measured in the water column using ADV. These elevated errors coincide with the bar crest region where wave breaking, strong turbulence, and flow separation are most pronounced, challenging the model’s ability to capture the mean flow structure. To provide a transparent overview of model performance, Table 4 summarises the RMSE data from Table 3, reporting the mean and range for each variable both including and excluding the reflection-affected station. When the *x = 53* m station is excluded, the mean RMSE for *η* across the remaining six stations (calculated from Table 3) is 0.05 m. The summary confirms the model’s high fidelity, with the largest errors confined to the bar crest region.

**Table 3:** Error metrics across selected transects (*x = 51*–63 m) for the SINBAD test case.

*x* (m)	*η* (m)	$U_{\textrm{fs}}$ (m s$^{-1}$)	$U_{\textrm{nb}}$ (m s$^{-1}$)	$U_{\textrm{ut,ADV}}$ (m s$^{-1}$)	$U_{\textrm{ut,ACVP}}$ (m s$^{-1}$)
51.0	0.07	0.13	0.22	0.06	0.07
53.0	**0.08**	0.14	**0.28**	0.16	0.11
55.0	0.06	0.09	0.15	0.21	0.09
56.6	0.03	0.14	0.07	**0.34**	0.09
58.0	0.03	0.14	0.13	0.18	0.09
60.0	0.04	**0.15**	0.08	0.26	**0.13**
63.0	0.04	**0.15**	0.16	0.13	0.12

**Table 4:** Summary of RMSE metrics derived from Table 3.

Variable	Mean RMSE	Range of RMSE	No. of stations
*η* (m)	0.06	0.03–0.08	7
$U_{\textrm{fs}}$ (m/s)	0.13	0.09–0.15	7
$U_{\textrm{nb}}$ (m/s)	0.16	0.07–0.28	7
$U_{\textrm{ut,ADV}}$ (m/s)	0.21	0.06–0.34	7
$U_{\textrm{ut,ACVP}}$ (m/s)	0.10	0.07–0.13	7

Preprocessing step was done by aligning the modelled and measured data before computing the error metrics.

### Phase resolving near bed velocity

**Fig. 6:**
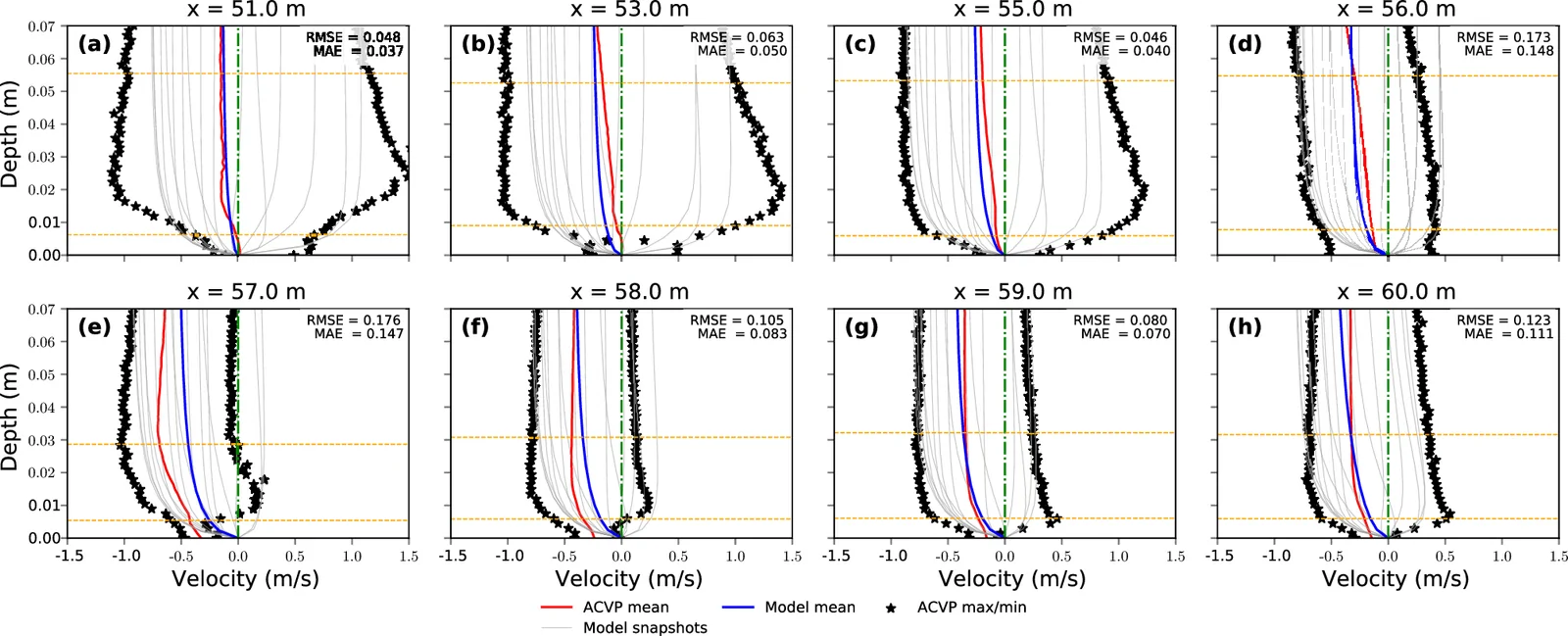
Vertical profiles of calculated and measured phase-averaged horizontal velocity between *z = 0.0-0.1* m for SINBAD test. The grey lines show the computed results. The green-dashed lines present zero velocity; Red and blue lines are observed and modelled mean flows, respectively; dashed yellow lines represent free-stream (top) and near-bed levels (bottom) for each location; and black stars show the measured velocities from the ACVP.

To examine the transformation of oscillatory velocities from the free-stream to the near-bed region, Fig. 6 presents vertical profiles of instantaneous horizontal velocity between *z=*0 and 0.1 m at several measured locations in the SINBAD test. Grey lines show computed velocity profiles at 0.1 s intervals over a wave cycle, and symbols denote measured peak onshore and offshore velocities.

In the shoaling region (51–53 m), a well-developed wave boundary layer is evident, with onshore velocity overshoots exceeding those offshore, indicating enhanced near-bed skewness. Measurements show a boundary-layer thickness of 2–2.5 cm, whereas model estimates are around 1–1.5 cm, which is likely due to the overestimated near-bed turbulence viscosity. Between the breaking and plunging points (53–55 m), the wave-dominated flow is evident, with strong oscillatory flow controlling the near-bed circulation. In the offshore side of the bar, the magnitudes of the offshore velocity decrease as the wave energy dissipates. This contrasts with the bar crest region, where the offshore velocity peaks at approximately -0.8 m/s due to the presence of undertow. As a result, skewness decreases compared to the shoaling zone, and the onshore/offshore durations become nearly equal, reducing asymmetry. In the near-bed region, the boundary layer height is reduced to approximately 1–1.5 cm. However, the onshore flow remains stronger than during the offshore period, indicating a wave-dominated oscillatory regime. From the plunging to splashing region (*x=56*–58 m), wave energy is substantially reduced, and the magnitude of the oscillatory velocities falls within *± 0.5* m/s at the free-stream. The strong undertow pushes the oscillation to the offshore side during the majority of the wave periods, or even totally offshore, directed at 57–58 m. The boundary layer is compressed to within 1 cm of the bed. Velocity profiles become nearly depth-uniform, suggesting very weak wave oscillatory pressure forces in comparison with those driving the undertow current. However, measured onshore overshoots are anomalously large, likely reflecting downward penetration of the surface jet^19^ during the passing of the wave bore at the wave crest, which produces elevated near-bed skewness despite modest asymmetry.

In the inner surf zone (58–60 m), oscillatory velocities at the free-stream level begin to recover, and the influence of the undertow gradually becomes less apparent. Near the bed, the onshore and offshore durations again become comparable within the boundary layer, resulting in reduced skewness and asymmetry. The measured maximum onshore velocity is persistently larger than that at maximum offshore phase, which is likely due to the presence of near-bed streaming that is not included in the model.

The model comparison against experimental data shows reasonable agreement. Within the shoaling and breaking zones, the model under-predicted the onshore flow magnitude due to the under-estimated wave height as shown in Fig. 2 and a lower value of near-bed friction. However, the reduction in the onshore velocity magnitude and the evolution of the wave nonlinearity from breaking into the surf zone are reproduced by the model, as shown by the identical pattern with the experiment.

**Fig. 7:**
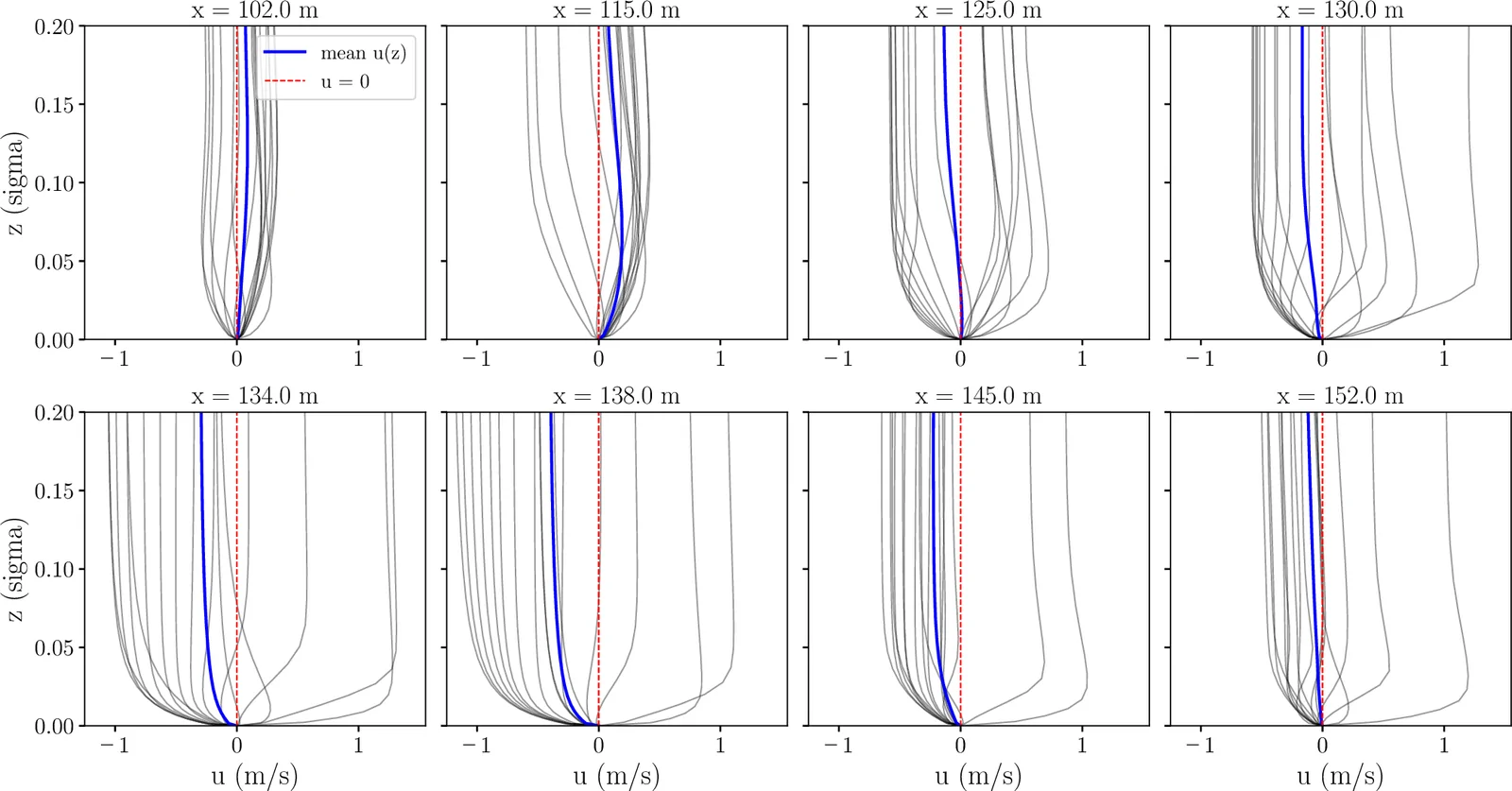
Calculated phase-resolved horizontal velocity vertical profiles at *z = 0.0-0.2* m above the bed for the LIP11-1C test under high wave condition. Grey lines show individual phase over one wave cycle. Red and blue lines are observed and modelled mean flows, respectively.

**Fig. 8:**
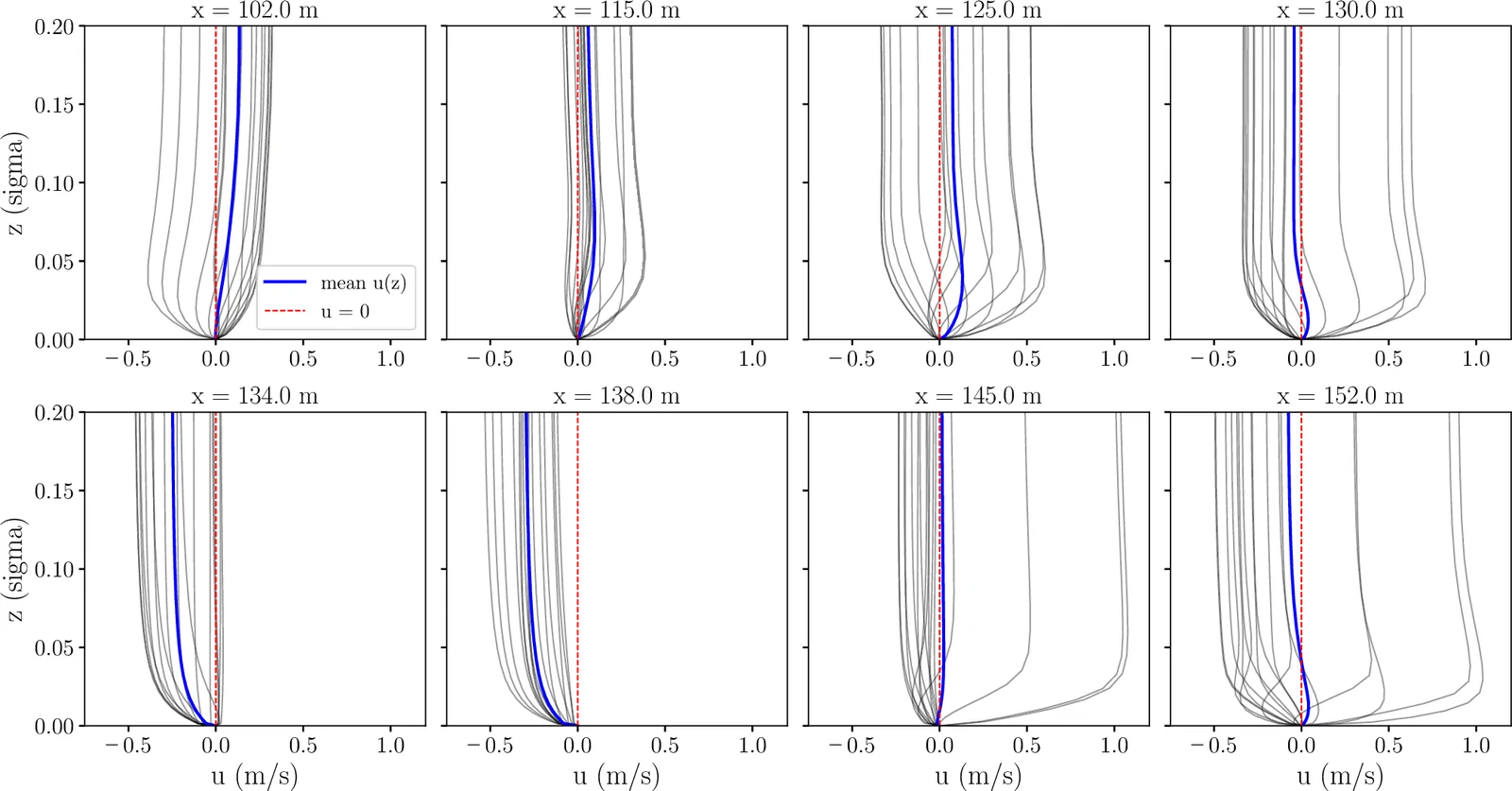
Similar configurations as in Fig. 7, but for small wave condition.

To examine the nonlinearity within the WBL under different wave conditions, Figs. 7 and 8 present the vertical profiles of the horizontal velocity computed in the LIP11-1C. In particular, Fig. 7 depicts the velocity under a representative large wave of the random wave group within 0.20 m above the bed across the shore, from *x=60* to *x=150* m. The corresponding wave period is approximately 4 s, and the wave height is about 1.10 m. In each subplot, sixteen grey curves represent profiles at evenly distributed wave phases over one wave cycle. The thick blue line denotes phase-averaged velocity profiles over depth $\bar{\textrm{u}}$, and the red line indicates the zero velocity level *u=0.0* m/s. Figure 8 shows the corresponding velocity profiles in a representative small wave inside the WBL, with a height of the wave of 0.20 m and a period of 5.30 s.

Figure 7 shows that the vertical velocity profiles exhibit patterns broadly similar to those observed for the energetic plunging breaker in the SINBAD experiment. In the shoaling zone (*x<130* m), increasing wave height leads to a gradual intensification of near-bed oscillatory velocities. The wave-driven boundary-layer flow dominates this region, with a pronounced onshore-directed overshoot. The boundary layer is able to grow over each wave cycle and can extend up to approximately 0.75 m above the bed, consistent with theoretical predictions^37^ and previous laboratory and field observations^38,39^.

As wave breaking initiates at *x=130* m, the boundary layer is compressed to within about 0.20 m of the bed, and the vertical profiles become nearly uniform, similar to those shown in Fig. 6. Although onshore velocities remain larger than offshore velocities, their duration is short, producing substantial wave skewness. Unlike the plunging breaker in the SINBAD case, the spilling breaker in this case does not significantly alter the near-bed boundary-layer dynamics; consequently, the pronounced onshore overshoot seen in Fig. 6 is absent. The undertow in this case is also weaker than in SINBAD and is insufficient to drive the oscillatory flow entirely offshore through most of the surf zone (134–150 m). As a result, the oscillatory boundary-layer structure retains a broadly similar form across the surf zone, though with gradually decreasing magnitude due to the continuing breaking, particularly during the onshore phase.

In contrast to Figs. 7, 8 illustrates the wave boundary layer beneath a representative small wave. In the shoaling region (*x<134* m), the boundary layer remains shallow, typically less than 0.25 m, due to the low wave energy. A weak near-bed streaming current produces a slight onshore bias in the oscillatory flow. Following wave breaking (*x>134* m), the reduced wave energy results in very small oscillatory velocities, and the undertow drives the near-bed flow increasingly offshore. Immediately after the breaking (*x<138* m), the undertow is strong enough to overcome the weak wave-driven motion, pushing the oscillatory flows offshore throughout much of this region. However, farther inside the surf zone (*x>145* m), as the wave gradually recovers and shoals, the magnitude of the near-bed oscillations begins to increase, particularly during the onshore phase, and the near-bed overshoot progressively returns to pre-breaking levels. Notably, in this low-energy case, wave breaking only modestly suppresses the boundary layer thickness and leaves little discernible imprint on the vertical flow structure or on near-bed nonlinearity, unlike the clear signatures observed in Fig. 6.

From the above comparisons, it is evident that under energetic waves, plunging breakers exert strong influences on both the free surface and the free-stream velocities. When the plunging jet interacts with the boundary layer, the near-bed velocities are also noticeably modified. Under milder wave conditions, spilling breakers produce much weaker effects on the free-stream and near-bed motions. In both cases, however, the undertow acts throughout the entire water column, driving offshore-directed flow from the free surface down to the seabed. As a result, the local wave nonlinearity is altered accordingly.

### Time series of surface elevation and fluid velocities

**Fig. 9:**
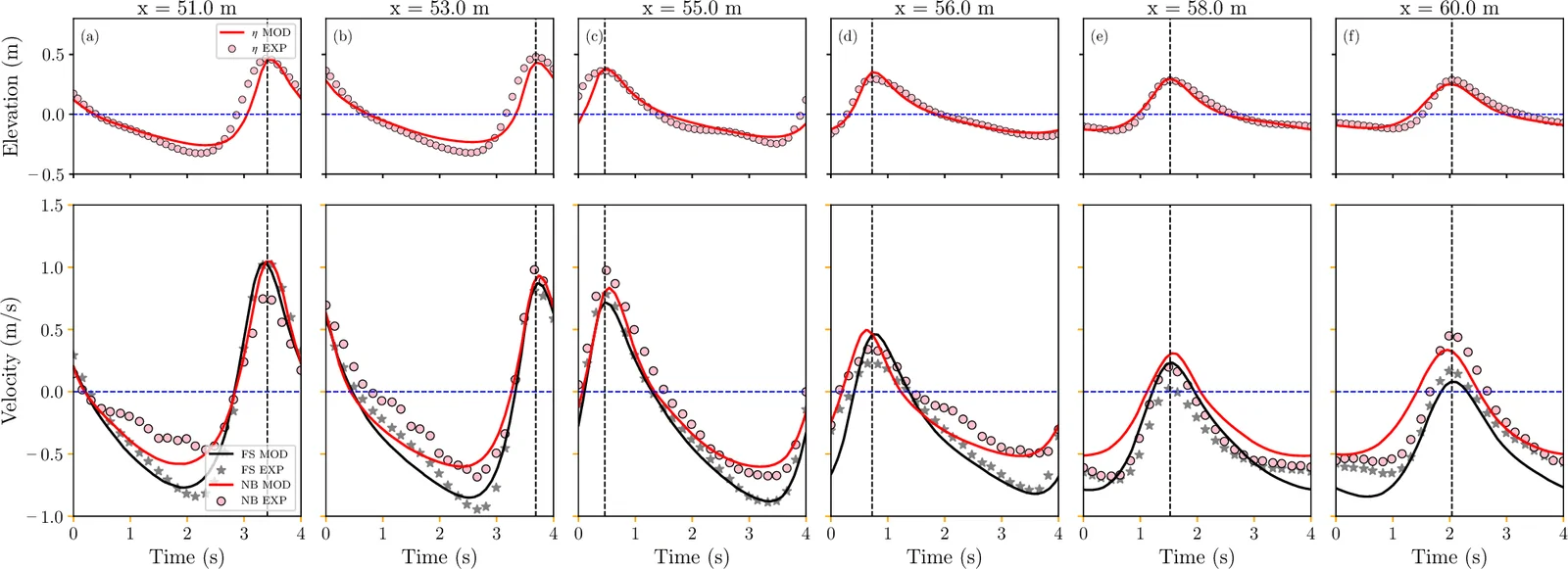
Comparison of instantaneous surface elevation, free-stream, and near-bed velocities against measurements at selected stations for SINBAD test.

To examine the relationship between surface dynamics and wave nonlinearity at both the free-stream level and the near-bed region, Fig. 9 shows the computed and measured instantaneous free-surface elevations and oscillatory velocities over a wave cycle at selected stations in the SINBAD experiment. The velocity time series are plotted at the corresponding vertical levels indicated in Fig. 6. The surface waveform evolves from a sharp-crested, Stokes-like form in the shoaling zone (51 m) to a sawtooth pattern shoreward of the breaking point (>55 m). The free-stream velocity follows these changes closely, transitioning from a nearly sinusoidal shape to a strongly asymmetric sawtooth-like signal around the breaking point (55–56 m). In the inner surf zone (>58 m), the wave gradually returns to a more Stokes-like form with an extended trough, and the free-stream velocity similarly evolves back toward a Stokes-type pattern, though shifted offshore due to the undertow. In contrast, the near-bed velocities at these locations maintain a broadly similar Stokes-like shape, exhibiting only modest variations in nonlinearity. The model captures these spatial transformations with good fidelity, reproducing both the magnitude and phase of the measured wave-induced oscillations.

### Phase-leading inside WBL

**Fig. 10:**
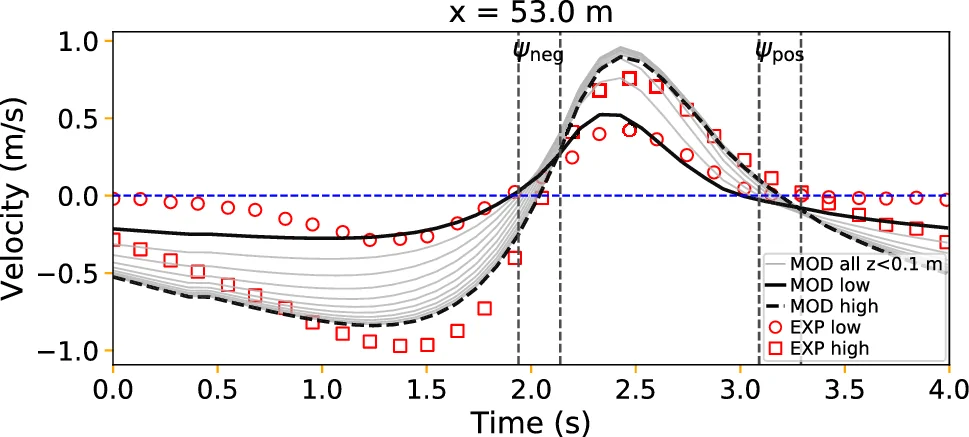
Phase lead for positive and negative half-cycles, describing down-crossing (crest-to-trough) and up-crossing (trough-to-crest) in SINBAD test, respectively.

In addition to the time-series analysis, the phase shift between free-stream and near-bed velocities provides an additional indicator of how wave nonlinearity transforms with depth. Using both the measured and computed velocities, the phase lead within the wave boundary layer (WBL) relative to the free-stream level is evaluated for the SINBAD and LIP11-1C tests. Following the previous study^40^, Fig. 10 shows the phase lead for negative and positive half-cycles based on the first harmonic component of the velocity signal, separated into positive (down-crossing; crest-to-trough) and negative (up-crossing; trough-to-crest) half-cycles.

**Fig. 11:**
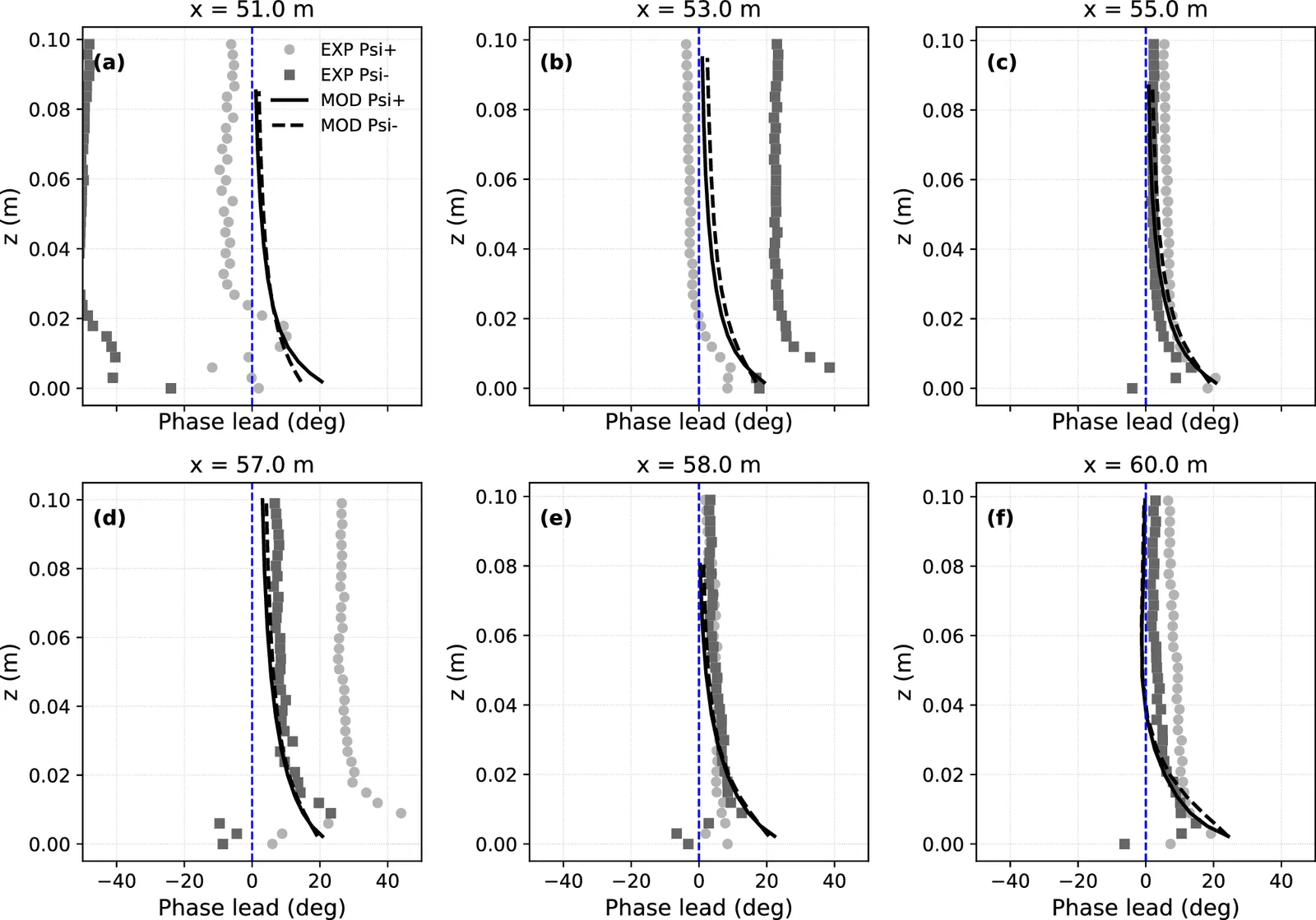
Model-data comparison of the phase lead between free-stream and near-bed velocity for the SINBAD test. Each subplot shows phase lead (degrees) as a function of depth at the selected cross-shore positions. Symbols denote experimental, while lines are simulation results. The phase lead is divided into positive and negative half-cycles, describing crest-to-trough and trough-to-crest, respectively.

Figure 11 compares the modelled and measured phase shift across the bar region for the SINBAD test. At each station, the phase lead is shown as a function of depth, with symbols representing measurements and lines representing the model results. In the shoaling zone (*<51* m), the measurements indicate a small ($<10^o$) positive phase shift aloft during up-crossing, and a larger ($>25^o$) negative shift close to the bed during down-crossing. The model captures the positive near-bed shift during up-crossing but does not reproduce the measured negative shift during down-crossing, implying an over-prediction of trough duration near the bed. At the breaking point (53 m), the observations show nearly zero shift during up-crossing but a $20^o$–$25^o$ positive shift during down-crossing, consistent with a shortened onshore and prolonged offshore phase. Shoreward, in the surf zone, both up- and down-crossing shifts become positive, with larger values for up-crossing. The model reproduces these patterns reasonably well, with some under-prediction of the up-crossing phase lead at 57 m.

**Fig. 12:**
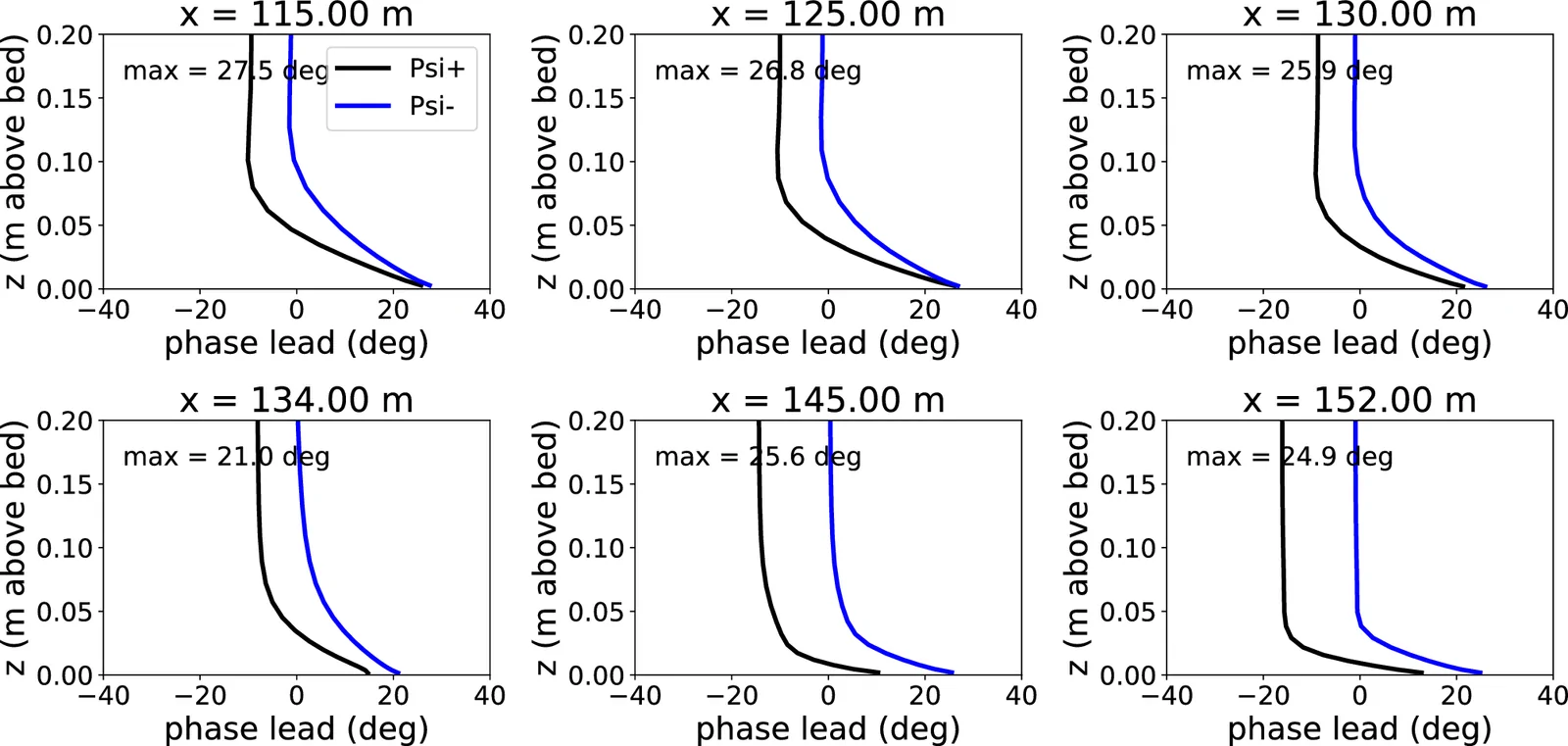
Vertical distribution of the phase lead as a function of the depth fore LIP11-1C case, with the focus on the 0.2 m above the bed. Each panel exhibits positive and negative half-cycles of the phase lead (in degrees) at six selected positions. Black and blue lines denote positive and negative half-cycles of the phase lead.

For the smaller-wave LIP11-1C test (Fig. 12), the computed phase lead also falls within a similar $20^o$–$30^o$ range across the bar region (115–145 m). The largest phase lead ($\approx 40^o$) occurs offshore at 65 m, after which the magnitude decreases gradually from 112 to 138 m before increasing again over the bar trough (145–160 m). Although no measurements are available for this case, the computed phase lead is consistently larger during up-crossing than down-crossing, particularly in the surf zone, similar to the SINBAD results.

Across both tests, the model predicts an almost uniform phase lead of $20^o$–$25^o$ over the inner bar. In the simulations, the waves in this region are dominated by a narrow frequency band near the peak period, and the near-bed flows are fully turbulent. Prior studies of oscillatory boundary layers show that the phase shift between the wave-induced pressure gradient and near-bed velocity is largely governed by the dominant wave period^13,41,42^. Because the peak period varies only weakly over the bar, the model yields a nearly constant phase lead. The SINBAD measurements, however, exhibit a broader range of phase shifts due to strong wave breaking and enhanced nonlinearity. In the shoaling zone, the phase lead is primarily governed by surface-wave nonlinearity, whereas wave breaking tends to amplify the up-crossing (trough-to-crest) phase lead relative to the down-crossing, as seen in both experiments and simulations.

### Wave skewness and asymmetry

#### Wave nonlinearity across the beach

**Fig. 13:**
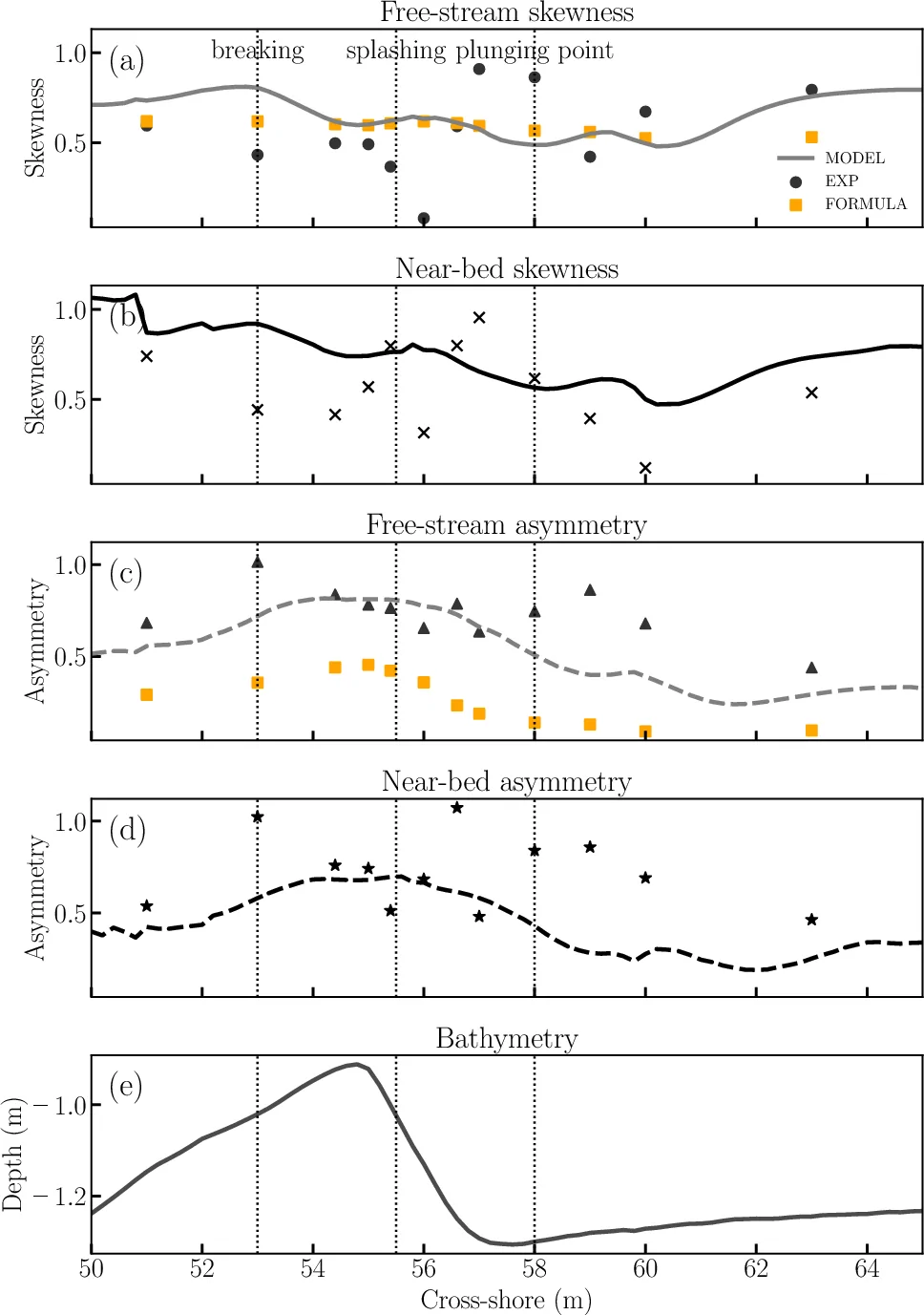
Predicted and measured free-stream and near-bed velocity skewness (**a**, **b**) and asymmetry (**c**, **d**) comparison at the SINBAD test. Symbols are measured-based *Sk* and *As*, while lines are simulated-based.

Figure 13 presents the computed wave skewness *Sk*(a–b) and asymmetry *As*(c–d) for both free-stream and near-bed velocities, compared with values estimated from the SINBAD measurements. The observations show that both *Sk* and *As* reach high values (0.6–0.7) on the offshore face of the bar, where wavelength shortening produces sharper-crested, forward-leaning waves (Fig. 9a). As the wave breaks at 53 m, *Sk* drops below 0.5 at both levels, while *As* exceeds 1.0, reflecting reduced oscillation magnitude and increased down-crossing phase lead (Figs. 9b; 11b). The low *Sk* persists through the plunging point (55 m), whereas *As* gradually relaxes toward its pre-breaking value. These changes correspond to the evolution from a steep, asymmetric waveform to a sawtooth shape with a pronounced onshore peak and flatter trough (Fig. 9c), and to the shift toward stronger up-crossing phase lead (Fig. 11c).

Shoreward of the plunging point, over the bar crest, free-stream skewness continues to decrease due to the reduction in oscillation amplitude, especially the onshore peak (Fig. 9d). Near the bed, however, the offshore peak weakens more strongly, resulting in an increase in *Sk*. The associated asymmetry remains similar to that at the breaking point. Once the plunging jet penetrates the water column and the splashing bore forms in the inner surf zone ( 58 m), *Sk* at both levels increases rapidly to values exceeding 1.0 before decaying shoreward. This growth is driven largely by the strong undertow in SINBAD, which suppresses the onshore velocity peak and shifts the oscillatory motion offshore while maintaining a near-uniform vertical profile (Figs. 9e–f and 6f–h). In contrast, *As* remains elevated (*>0.7*) in this region due to the presence of splashing bores that persist into very shallow water (*>63* m).

Comparison with measurements shows that the modelled skewness at both levels agrees reasonably well prior to the breaking point (53 m). However, the model does not reproduce the sharp reduction in *Sk* and the rapid increase in *As* at the breaking point because the plunging-breaking are absent from the breaking formulation. Within the breaking region (53–55 m), the model does capture the reduction in *Sk* but does not show the observed recovery over the bar crest. For the same reason, surface-elevation skewness is over-predicted in this zone. In the inner surf zone (*>58* m), the modelled skewness aligns reasonably well with measurements for both free-surface elevation and free-stream velocity. The predicted asymmetry agrees with the observations in the shoaling and breaking regions but decreases too rapidly shoreward of the splash point, partly due to the absence of splashing rollers in the model.

For comparison purposes, the estimated skewness and asymmetry at the free-stream level based on the empirical formula^7^ are also shown in (a) and (C). It can be seen that the estimated *Sk* closely follows the present model results, missing the plunging breaker effects in the measured data similarly as the 1DV model. The corresponding *As*, however, are fairly underestimated compared with the model results and measurements.

**Fig. 14:**
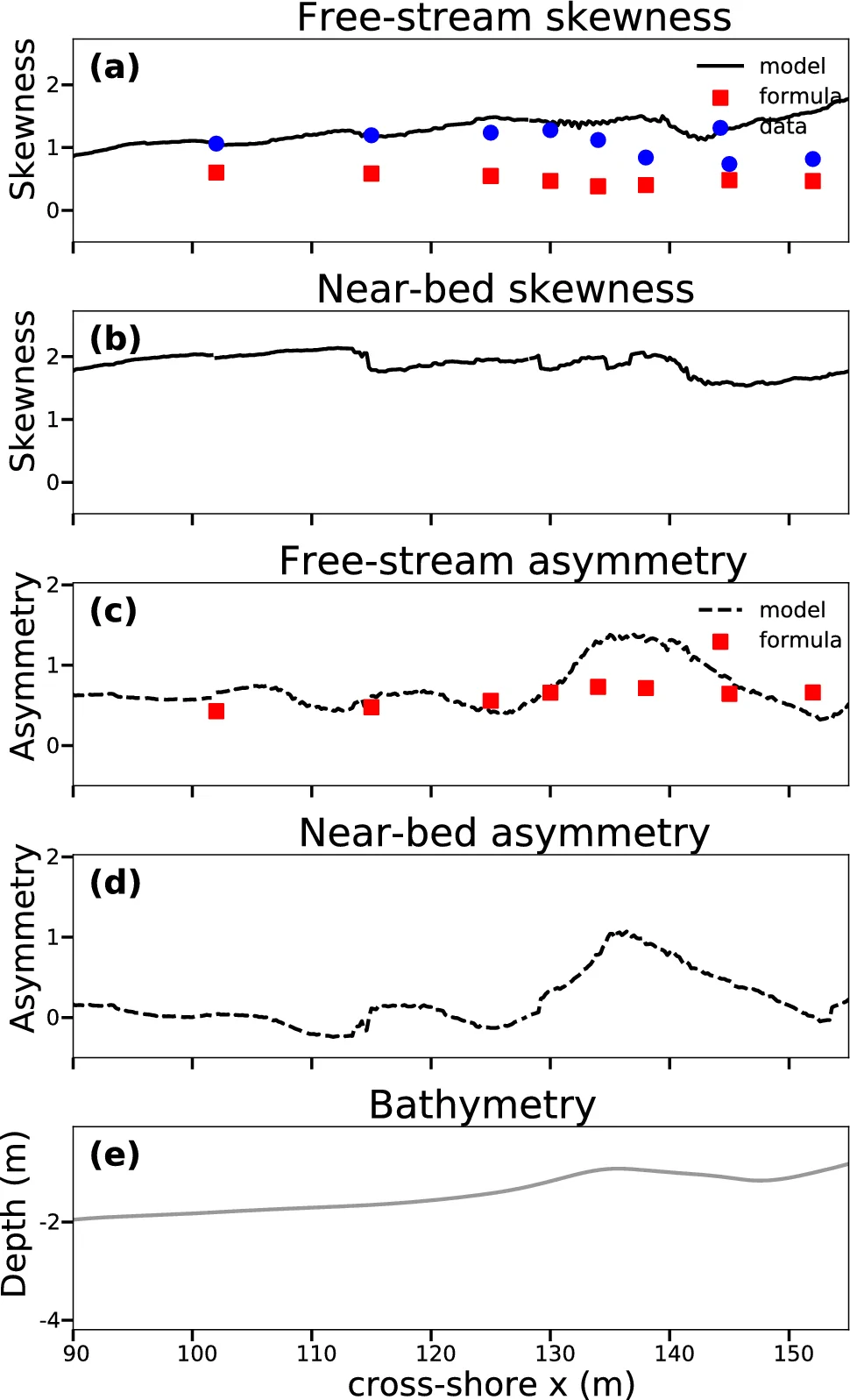
Predicted and measured free-stream and near-bed velocity skewness (**a**, **b**) and asymmetry (**c**, **d**) comparison at the LIP11-1C test. Symbols are measured-based *Sk* and *As*, while lines are simulated-based.

The computed *Sk* and *As* for the LIP11-1C test at the free-stream level are shown in Fig. 14. As in the previous section, the results are compared with values estimated using empirical analytical formulations^7^. In the offshore region (60–-125 m), the model agrees with the measurements, with *Sk* values exceeding 1.0. Approaching the breaking point, both measured and simulated *Sk* increase to a maximum of about 1.0 and then gradually decrease inside the surf zone. Model–data agreement is very good up to the bar trough. Shoreward of the trough, however, the model overpredicts *Sk*, consistent with the overestimation of setup noted earlier in Fig. 5. Overall, the agreement between the model and the observations is better than that obtained from the empirical expression of^7^.

The predicted free-stream *As* ranges between 0.3 and 0.4 seaward of the breaking point, followed by a sharp increase at breaking and in the outer surf zone. The asymmetry then decays gradually in the inner surf zone. Although no experimental data are available for *As* in this test, values estimated from empirical form^7^ are shown for comparison. The analytical estimates and model results agree well across the beach profile except in the very shallow region near the shoreline, consistent with the skewness behaviour discussed above.

In contrast to SINBAD, the LIP11-1C test exhibits a distinctly different distribution of intra-wave nonlinearity at the free-stream level, particularly within the surf zone. In the LIP11-1C case, the largest *Sk* and *As* are found near the breaking point, followed by a gradual decay shoreward, consistent with field observations under mild wave conditions^43,44^. In SINBAD, by contrast, the reductions in *Sk* and *As* occur at the plunging point, when the jet impacts the surface, rather than at the initial overturning location. Moreover, the splashing and prolonged roller in SINBAD induce a strong increase in *Sk* and maintain elevated *As* within the inner surf zone–behaviour not present under the spilling breaker of LIP11-1C.

It is noted that the present model is based on the eddy-viscosity concept for the breaking energy dissipation together with a breaking surface angle initiation approach. To simulate the plunging breaking process using such an approach is fairly challenging, which leads to discrepancies in the breaking region in the SINBAD test. However, the model is able to capture the overall wave skewness and asymmetry for both surface elevation and free stream velocity under the plunging breaker and the spiling breaker reasonably well as shown in Figs. 9, 13 and 14.

#### Wave nonlinearity over the depth

**Fig. 15:**
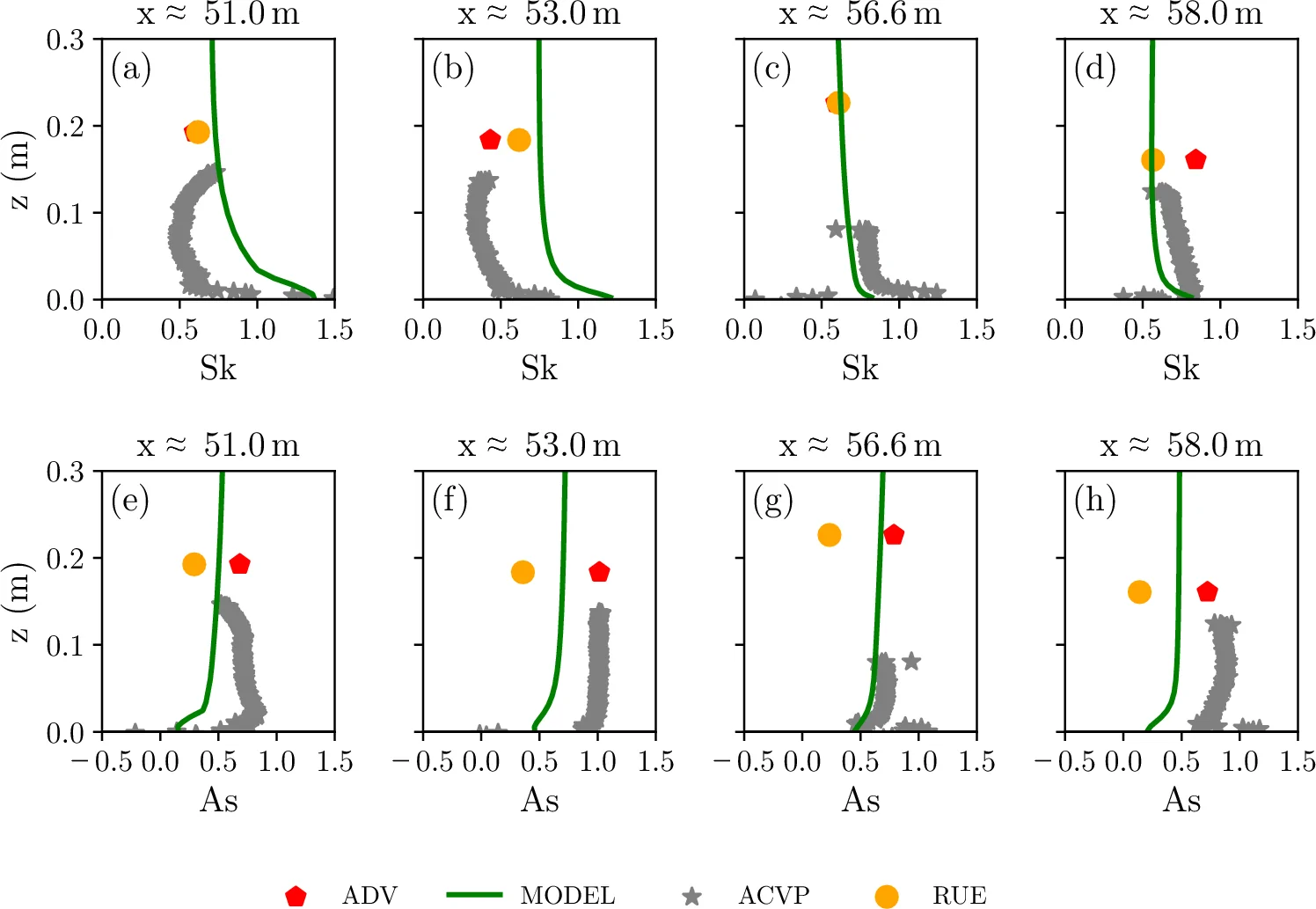
Vertical profiles of velocity skewness (**a**–**d**) and asymmetry (**e**–**h**) at breaking point (*x=53* m), splashing point (*x=56.6* m), and plunging point (*x=58* m) for the SINBAD test. lines present simulations, while symbols depict data (red and grey) and formula (yellow).

**Fig. 16:**
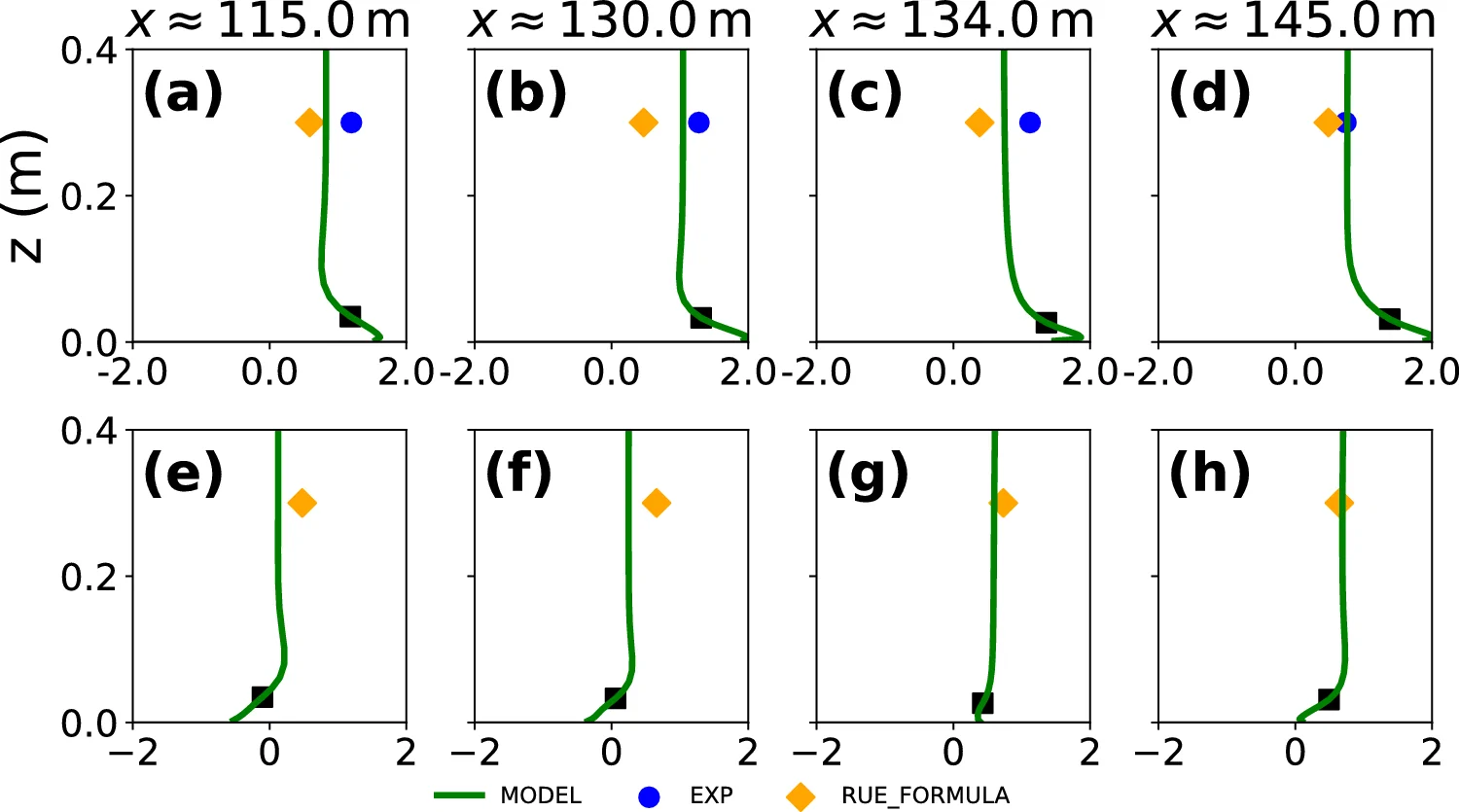
Vertical profiles of velocity skewness (**a**–**d**) and asymmetry (**e**–**h**) at shoaling (*x=115* m), breaking (within *x=130* and *x=134* m), and surf zones (*x=145* m) for the LIP11-1C test. lines present simulations, while symbols depict data (blue) and formula (yellow).

To examine the vertical structure of velocity skewness and asymmetry at different cross-shore locations, Figs. 15 and 16 present simulated third-order velocity moments together with measurements from the SINBAD and LIP11-1C experiments, as well as the analytical estimations^7^ (hereafter RUE).

In both data sets, $S_k$ and $A_s$ exhibit opposite trends with depth: $S_k$ increases downward, whereas $A_s$ decreases, consistent with earlier observations and theoretical arguments^13,40^. The wave boundary layer (WBL) progressively filters high-frequency harmonics and shifts the waveform from a combined skewed–asymmetric shape towards a predominantly skewed form, thereby enhancing near-bed nonlinearity^7,44^. Although this general pattern is evident for both plunging and spilling conditions, the magnitude and vertical distribution of the gradients differ markedly. In the plunging case, strong pressure penetration throughout the water column produces a relatively uniform vertical velocity profile (Fig. 6), consistent with previous observations of plunging bores^34,45^. As a result, while the compressed WBL still increases near-bed $S_k$, the intense plunging events tend to homogenise the velocity structure over depth. In contrast, under spilling breakers, the pressure field associated with the breaking front is weaker and insufficient to override the periodic wave motion. Consequently, substantial vertical variation emerges, with significant modulation of the velocity signal from the free stream down to the near-bed. These behaviours align with previous studies that describe the spatial evolution of higher-order velocity moments in shoaling and breaking waves^44,46^.

### Bi-spectrum analysis

Following previous studies^10,44^, bispectral analysis is performed to elucidate the mechanism of wave nonlinearity evolution over the water column and across the beach. The analysis focuses on detecting and quantifying nonlinear processes at the breaking point and in the surf zone (splashing and plunging points) under both high- and low-energy wave conditions. This method produces three plots: power spectral density (PSD), bicoherence, and biphase. The PSD describes how the variance (wave energy) is distributed over frequency. The bicoherence is the normalised magnitude of the bispectrum used to quantify the strength of quadratic phase coupling. The bicoherence contour varies from 0 to 1, with 0 meaning no coupling and 1 meaning full coupling. The biphase plots show the phase angle of the bispectrum at each triad ($\mathrm {f_1}, \mathrm {f_2},\mathrm {f_3}$), but more importantly, it is used to determine the type of wave distortion (*Sk* and *As*) associated with nonlinear processes. $\mathrm {0^o}$ indicates that the distortion is dominated by crest-trough skewness, while $\mathrm {90^o}$ means that the distortion is dominated by the front-back asymmetry. In this section, the bispectral analysis is applied at the breaking, splashing, and plunging points of the SINBAD test, and at the shoaling, breaking, and surf zone for the LIP11-1C test.

**Fig. 17:**
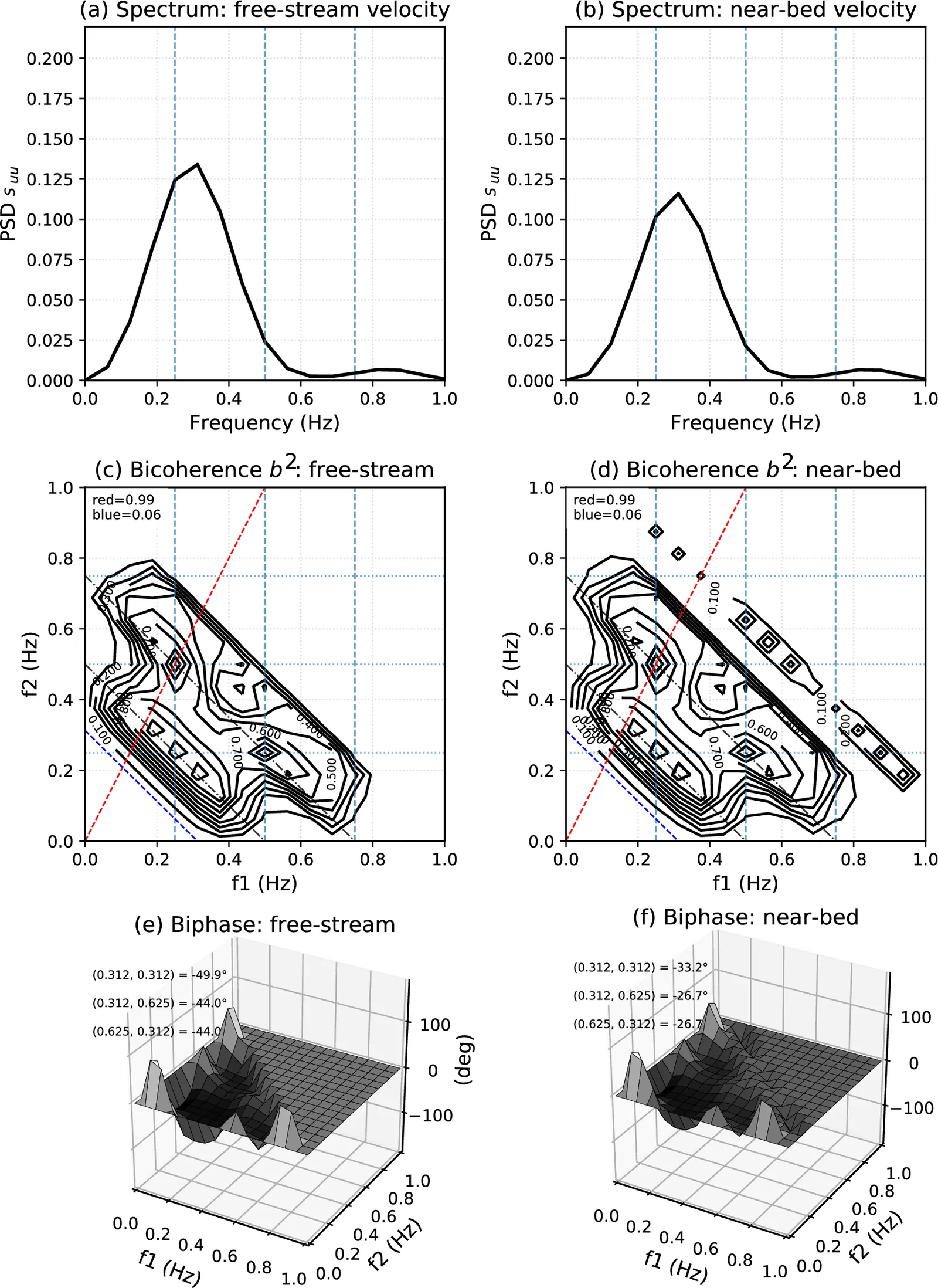
Bispectral analysis for SINBAD case: simulated-based power spectral density (**a**, **b**), normalised bicoherence intensity (**c**, **d**), and biphase (**e**, **f**) at the breaking point *x=53* m for free-stream (left) and near-bed (right) levels.

**Fig. 18:**
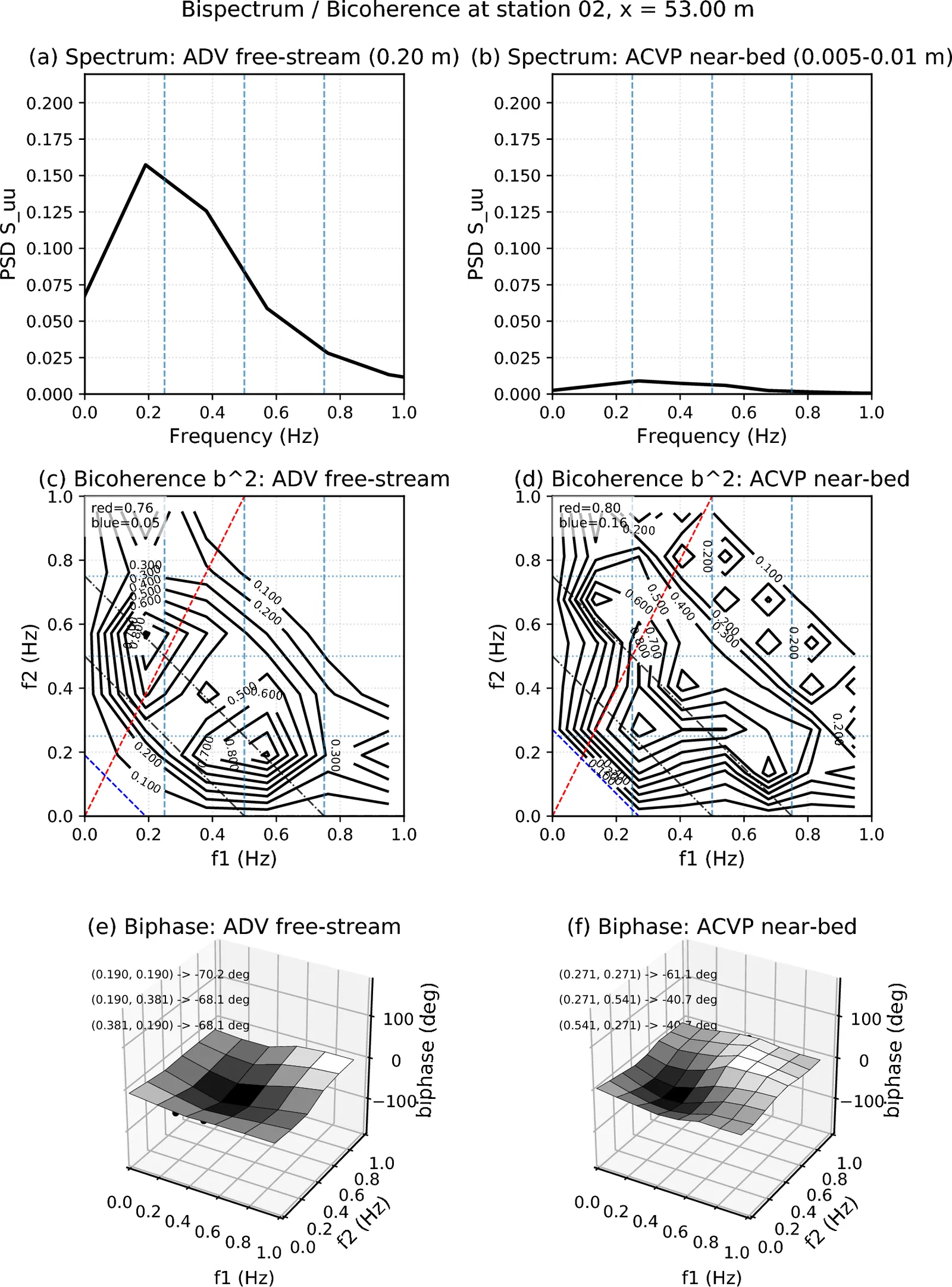
Bispectral analysis for SINBAD case: measured-based power spectral density (**a**, **b**), normalised bicoherence intensity (**c**, **d**), and biphase (**e**, **f**) at the breaking point *x=53* m for free-stream (left) and near-bed (right) levels.

**Fig. 19:**
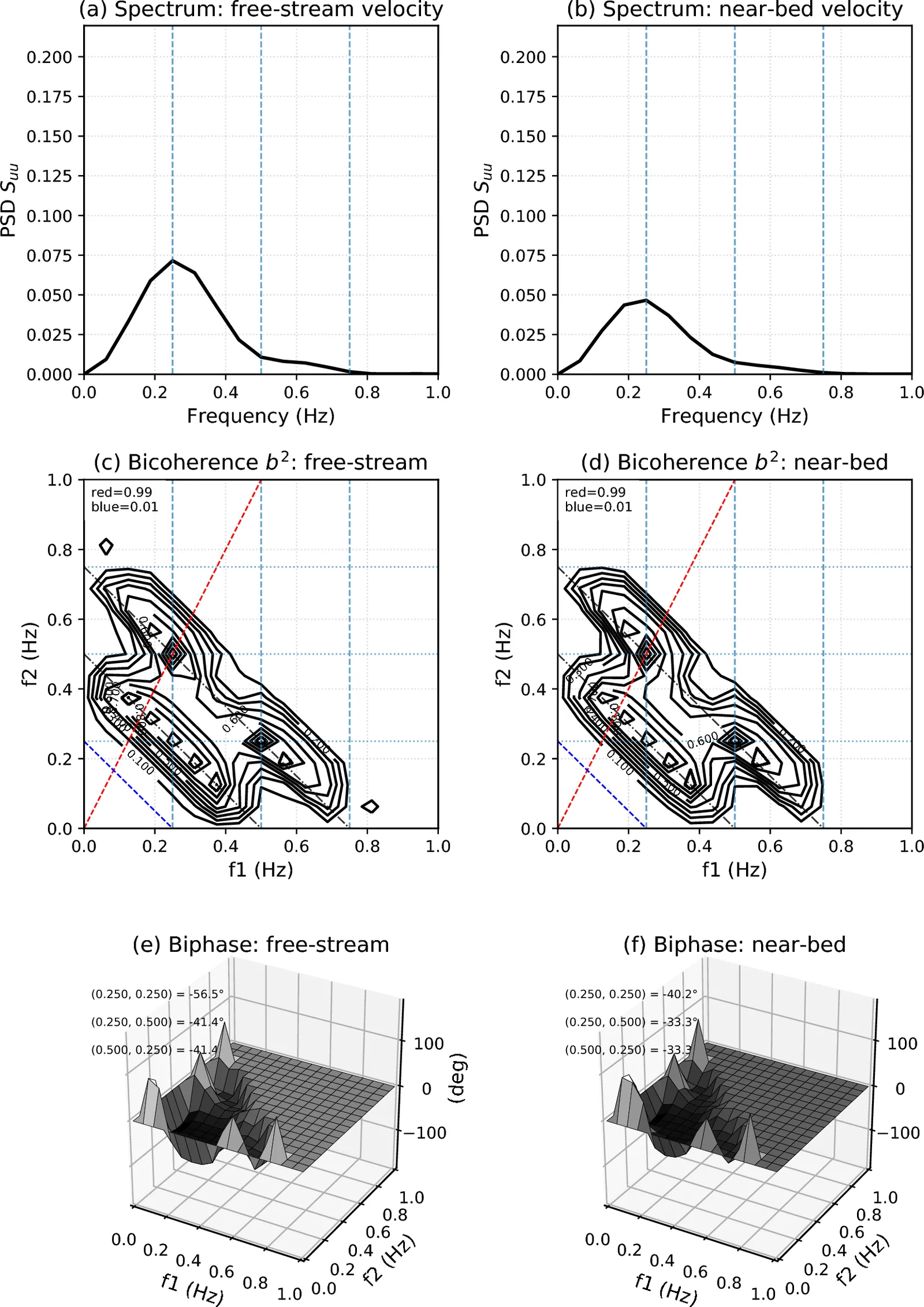
Bispectral analysis for SINBAD case: simulated-based power spectral density (**a**, **b**), normalised bicoherence intensity (**c**, **d**), and biphase (**e**, **f**) around the splashing point *x=56.6* m for free-stream (left) and near-bed (right) levels.

**Fig. 20:**
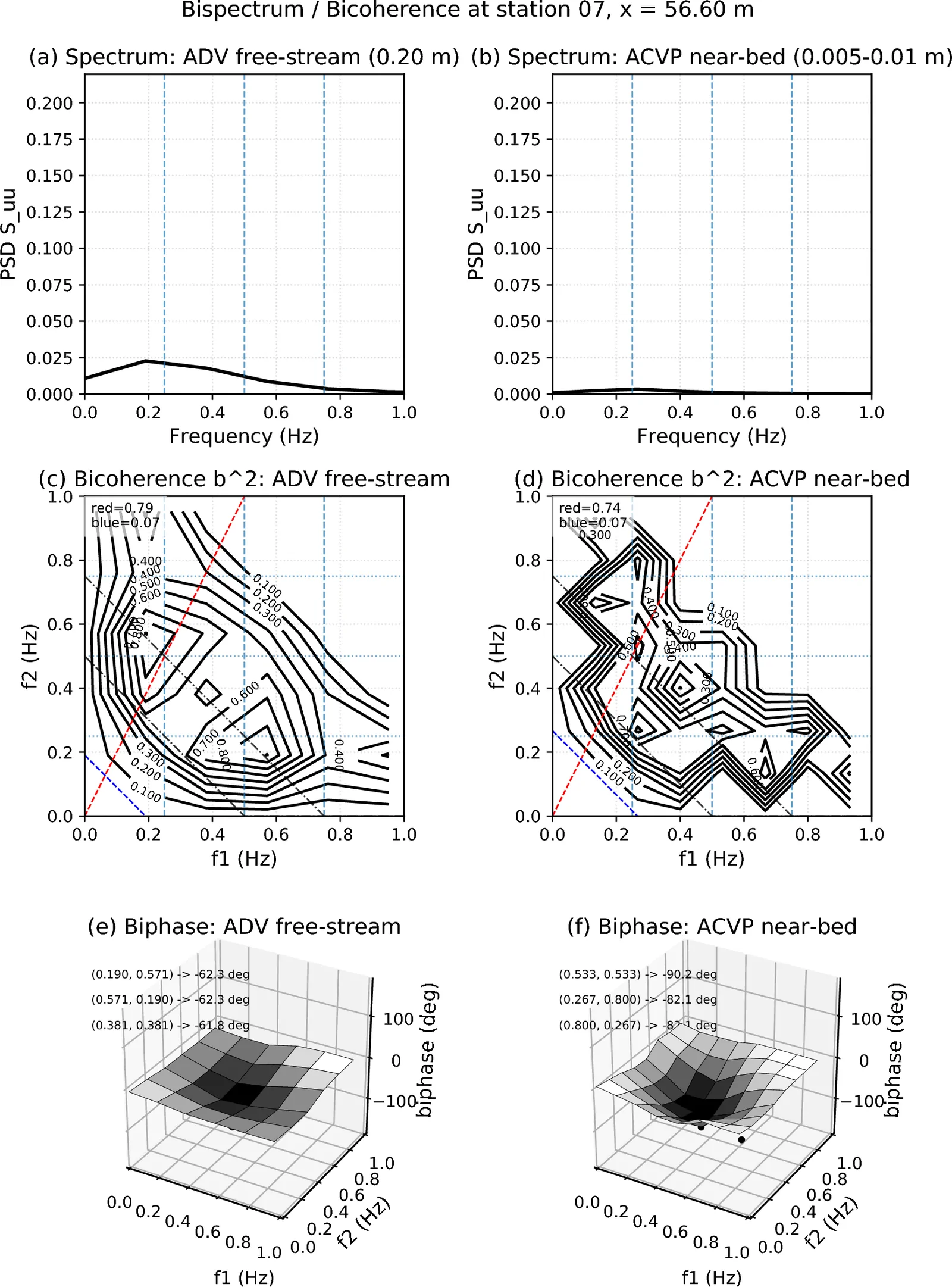
Bispectral analysis for SINBAD case: measured-based power spectral density (**a**, **b**), normalised bicoherence intensity (**c**, **d**), and biphase (**e**, **f**) around the splashing point *x=56.6* m for free-stream (left) and near-bed (right) levels.

**Fig. 21:**
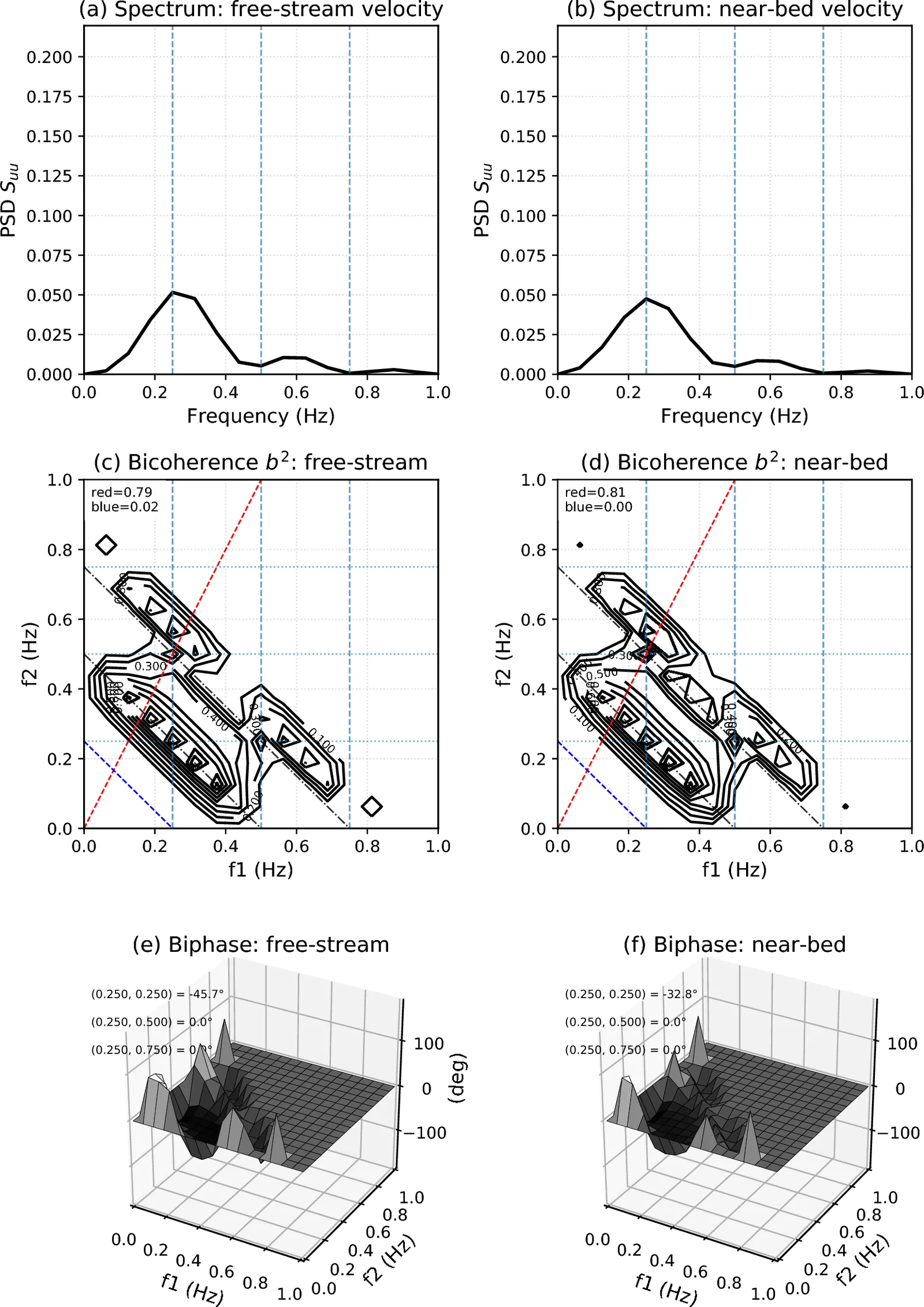
Bispectral analysis for SINBAD case: simulated-based power spectral density (**a**, **b**), normalised bicoherence intensity (**c**, **d**), and biphase (**e**, **f**) around the plunging point *x=58* m for free-stream (left) and near-bed (right) levels.

**Fig. 22:**
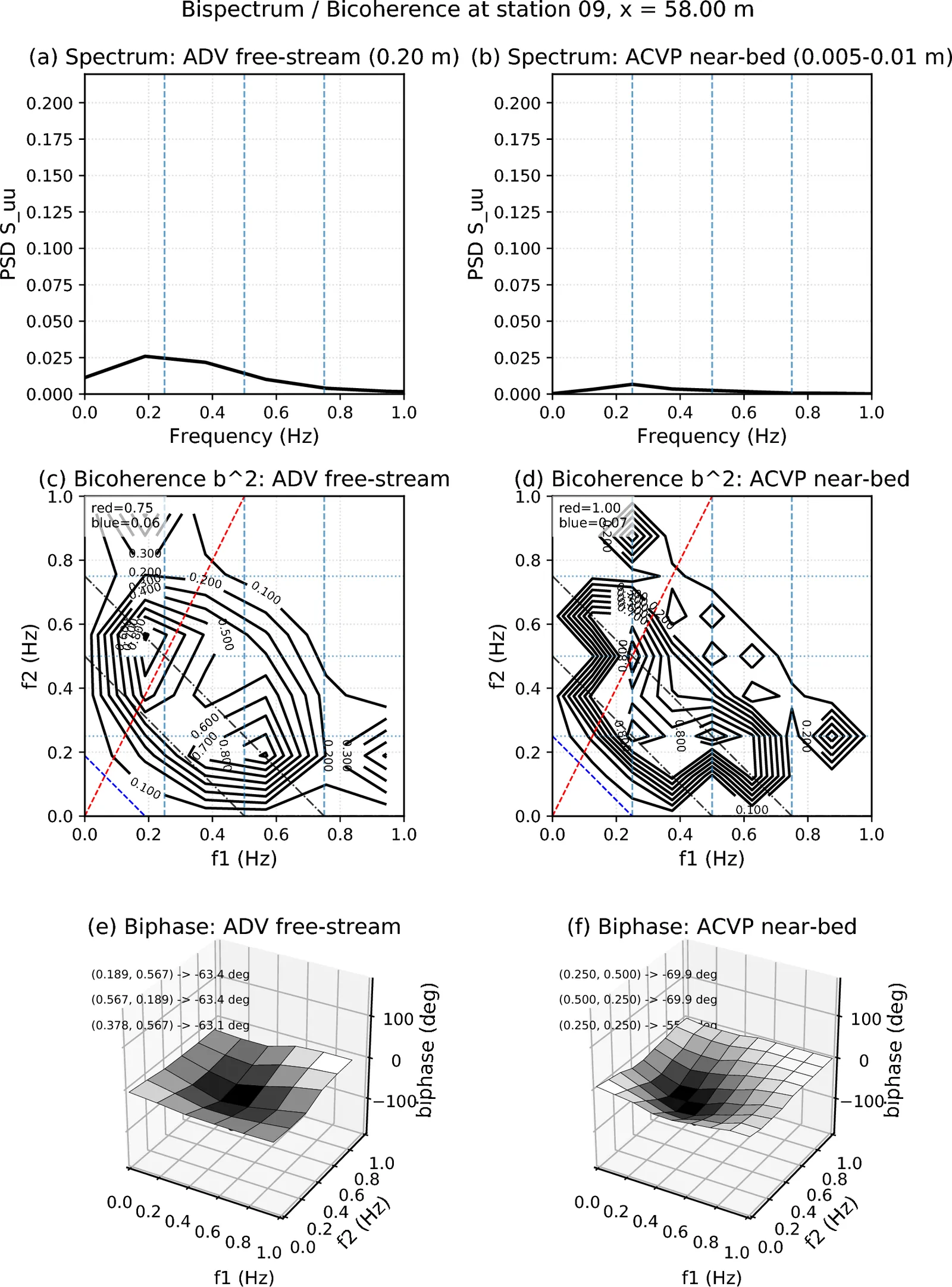
Bispectral analysis for SINBAD case: measured-based power spectral density (**a**, **b**), normalised bicoherence intensity (**c**, **d**), and biphase (**e**, **f**) around the plunging point *x=58* m for free-stream (left) and near-bed (right) levels.

Figures 17, 18, 19, 20, 21 and 22 exhibit model- and measurement-based PSD plots, bicoherence contours and 3D biphase plots at the free-stream and near-bed levels, at three different locations, breaking, splashing, and plunging points. Figure 17a and b present the simulated PSD at the SINBAD breaking point (*x=53* m) for free-stream and near-bed levels. Both PSD plots show a very sharp, narrow peak at about $\textrm{f}=0.25-0.31$ *Hz*, and then decay rapidly at higher frequencies. The PSD magnitude decreases only slightly from the free stream to the near-bed level. Inside the bicoherence contour, there are red and blue dashed lines. The red line represents pure harmonics of the triads $\textrm{f2}=2\textrm{f1}$ and $\textrm{f3}=\textrm{f1}+\textrm{f2}=3 \textrm{f1}$, while the blue dashed line shows the phase-locked interaction that feeds or modulates the main carrier $\textrm{f2}=\textrm{fp}-\textrm{f1}$. The harmonic components used in the coupling and biphase analysis are based on the PSD frequency, indicating the fundamental (f1) and higher harmonic components (f2 and f3). These harmonics present the self- or cross-coupling in bispectral analysis.

The bicoherence contours at the free-stream and near-bed levels indicate a strong, fully phase-locked self-harmonic interaction and weak sum-frequency mixing, as shown in Fig. 17c and d (red=0.99 and blue=0.07-0.05). The biphase values at the free-stream and near-bed levels are $-47^o$ and $-32^o$, describing a forward-leaning crest but not an extreme sawtooth. The experimental data show a larger PSD magnitude at the free stream but a very low one at the near-bed (see Fig. 18a, b). The strong 2$\textrm{f1}$ coupling remains, but it is weaker than in the simulation, with the $B^2 = 0.76$. Interestingly, the degree of phase-locking near the bed is larger than at the surface, highlighting greater sum-frequency mixing near the bed as seen in Fig. 18c, d. The biphase (Panels e-f) is more negative than the model, with values -70$^o$ and -50$^o$, describing a more sawtooth-like velocity shape than in the model. According to the bispectral analysis results, both simulations and measurements show a clear breaking signature.

At the plunging point, the PSD magnitudes for both the model and the experiment decrease significantly to about 0.02-0.07, compared to the breaking point as shown in Figs. 21 and 22. The predicted bicoherence contour shows strong pure-harmonic triads, with full coupling at the breaking point and almost no transfer of energy to the mixing frequency. The observed self-coupling interaction is smaller than the breaking point $\mathrm {b^2}=$0.79-0.72, which is a clear indication of phase scrambling by the breaking process. The model reproduces a moderate asymmetry signal, with a biphase magnitude ranging from $-40^o$ to $-50^o$. In contrast, a higher biphase magnitude is obtained from the measurement with the value -60$^o$ at the free-stream and -90$^o$ at the near-bed levels. A very strong phase-locking to plunging events describes the phase alignment of mixed harmonics with the main carrier and is also an indication of a plunging breaker.

The PSD magnitudes calculated by the model and measurements are in close agreement at the splashing point (Figs. 19 and 20). The energy level is smaller than at the plunging point, with values in the range 0.025 to 0.05. The computed bicoherence intensity based on experimental data shows a perfect triad phase-locking near the bed, with $\mathrm {b^2}=$1.0, which is bigger than the free-stream level where $\mathrm {b^2}$=0.76. Meanwhile, the bicoherence contour for the model results indicates a regular triad locking with equal $\mathrm {b^2}$ values of 0.8 at the surface and near the bed. In this position, the experiment shows a very strong negative biphase, $-70^o$, in both layers, with the maximum coherence near the bed. The simulation values range from $-45^o$ to $-32^o$, which still show the forward-leaning front with an asymmetrical slope. The experiment captures splashing effects in terms of self-coupling rather than the sum of the mixed frequencies, as detected by the near-bed signal.

Based on bispectral results, there are clear signs that the plunging breaker has a significant influence on the coupling of harmonic components. First, the PSD energy decreases in both the model and the experiment at the plunging point. However, the bicoherence shows strong self-coupling within the harmonic triads, with only weak transfer to mixed frequencies. The corresponding bicoherence values decrease in the experiment, indicating that the plunging event partially scrambles the phases of the harmonic components. The biphase becomes more negative (closer to $-90^o$) near the bed, consistent with the plunging bores affecting the bed and strongly distorting the waveform within the WBL.

The bicoherence reaches $\mathrm {b^2}= 1$ near the bed, which is larger than the free-stream with $\mathrm {b^2} = 0.76$, and also larger than the model with $\mathrm {b^2} = 0.8$0.8 at both levels. The associated perfect triad locking indicates a highly coherent harmonic structure in the near-bed velocities. The biphase depicts more negative values in the experiment than in the model. The breaking transforms the initially regular, bound wave-nonlinearity at the breaking point into a more bore-dominated regime.

**Fig. 23:**
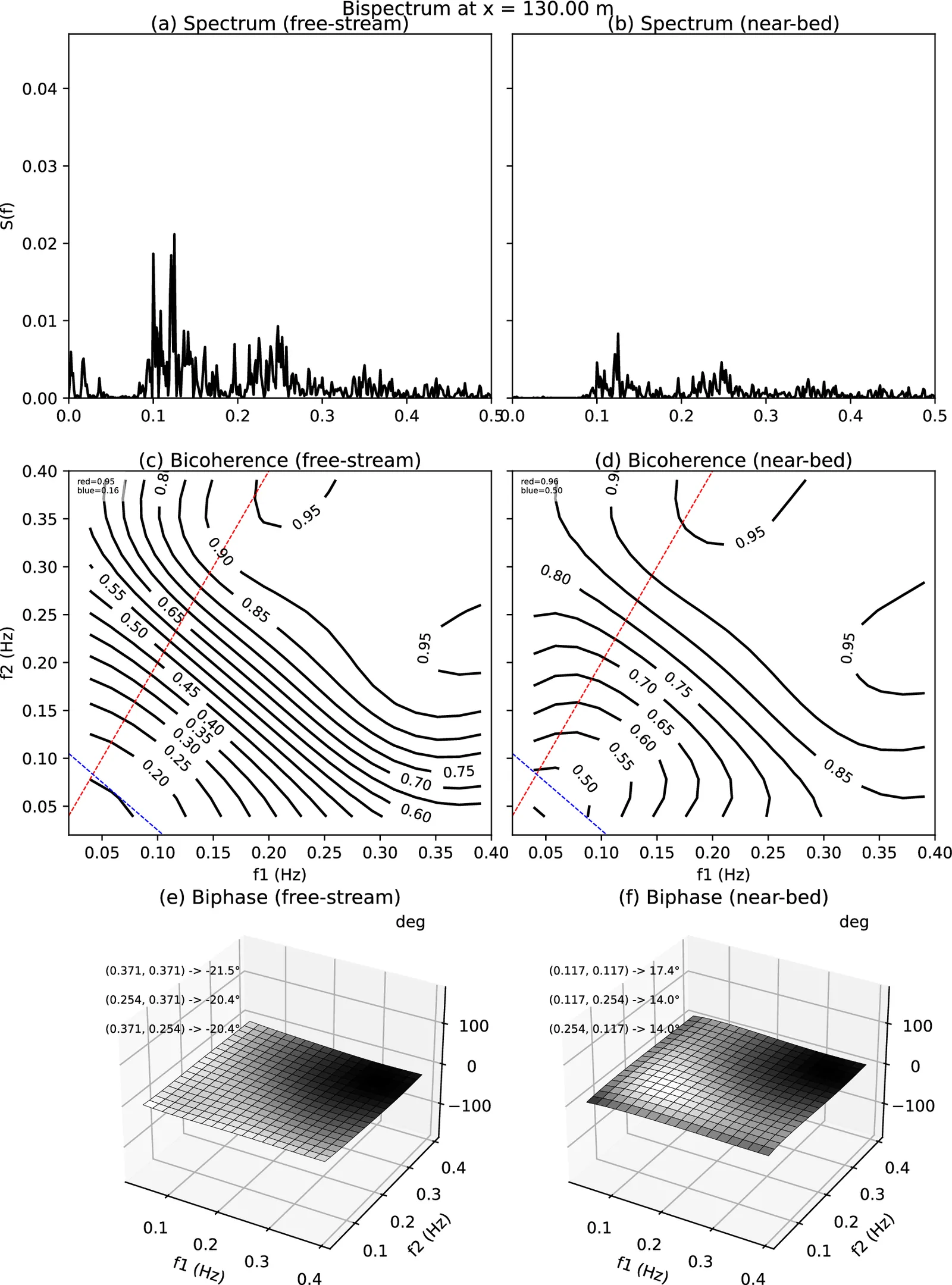
Bispectral analysis for LIP11-1C case: simulated-based power spectral density (**a**, **b**), normalised bicoherence intensity (**c**, **d**), and biphase (**e**, **f**) around the breaking point *x=130* m for free-stream (left) and near-bed (right) levels.

**Fig. 24:**
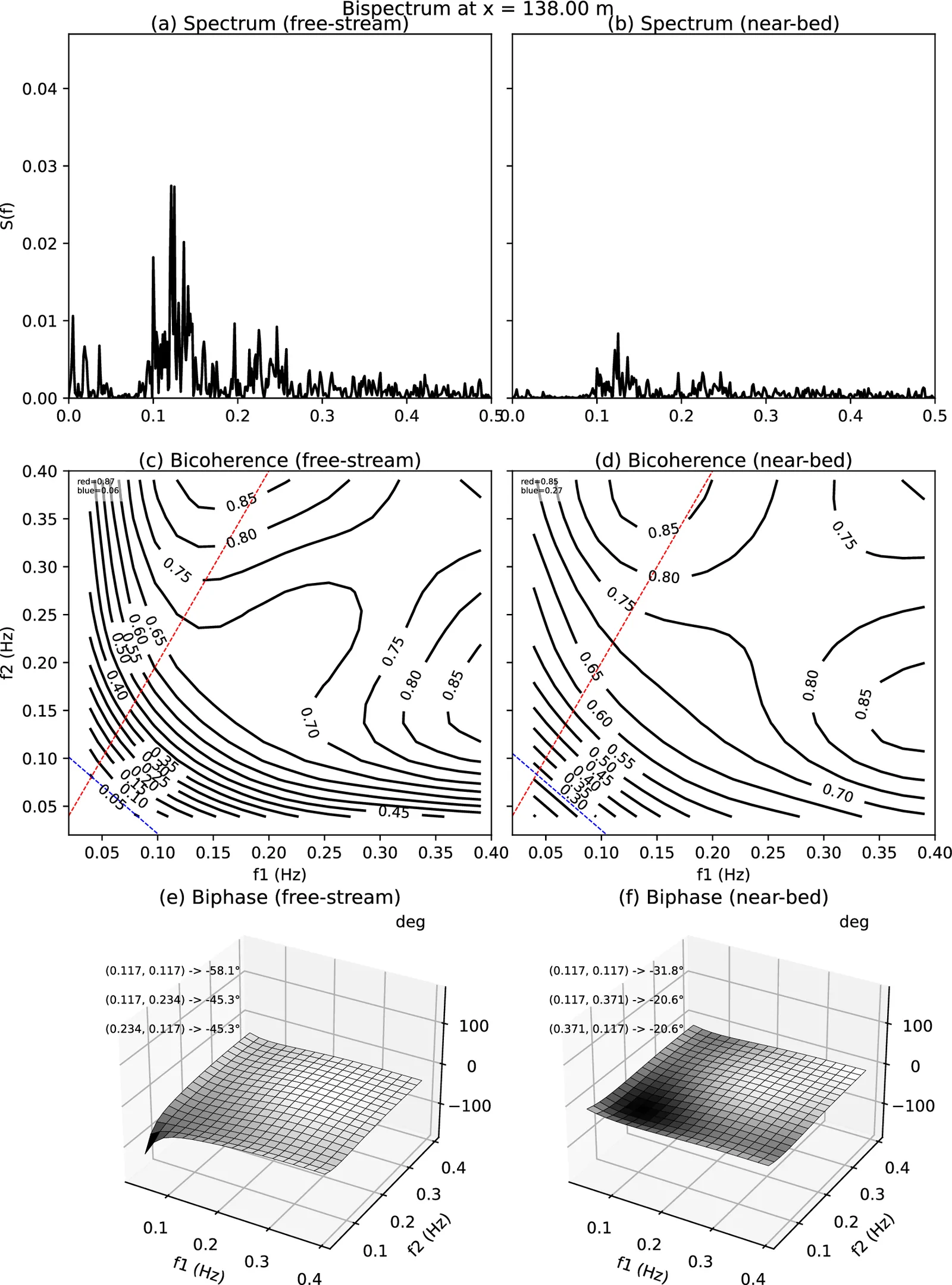
Bispectral analysis for LIP11-1C case: simulated-based power spectral density (**a**, **b**), normalised bicoherence intensity (**c**, **d**), and biphase (**e**, **f**) around the outer surf zone *x=138* m for free-stream (left) and near-bed (right) levels.

**Fig. 25:**
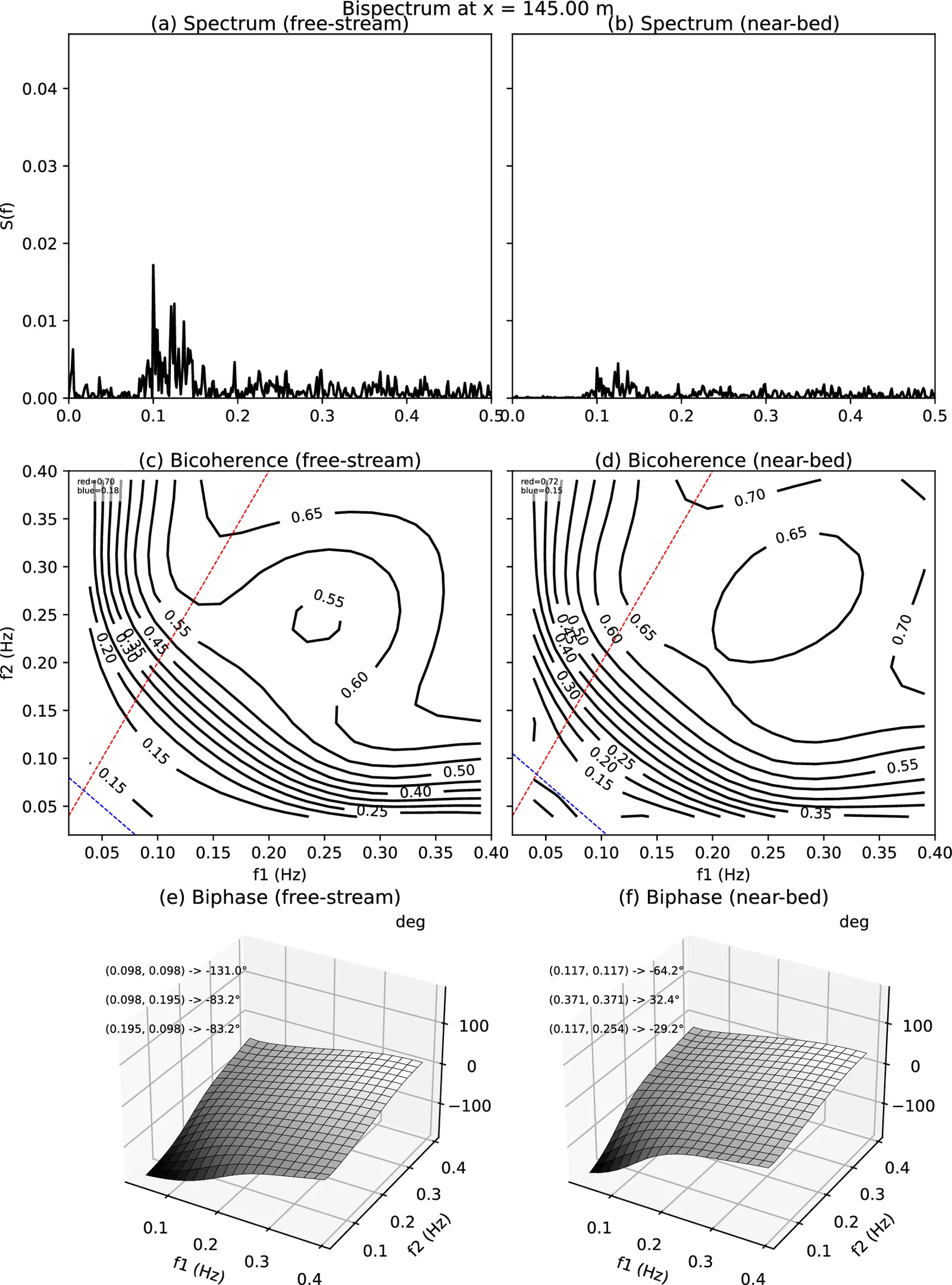
Bispectral analysis for LIP11-1C case: simulated-based power spectral density (**a**, **b**), normalised bicoherence intensity (**c**, **d**), and biphase (**e**, **f**) around the inner surf zone *x=145* m for free-stream (left) and near-bed (right) levels.

Bispectral analysis is also conducted for the LIP11-1C test. Figs. 23, 24 and 25 present PSD, bicoherence, and biphase plots at the free-stream and near-bed levels. Three positions at *x=130, 138, 145* m are used for the analysis: breaking, outer surf zone, and inner surf zone.

The PSD plot at the free-stream level shows a distribution of energy around the peak frequency at *0.12-0.14 Hz* and a minor secondary bump at 0.25*Hz* and *0.35-0.4 Hz* (Fig. 23a). The structure remains near the bed, but its magnitude decreases by more than a factor of two due to depth-induced attenuation as shown in Fig. 23b. The corresponding self-coupling is high for both the free-stream and the near-bed, with $\textrm{b}^2 = 0.95-0.96$. This means that the core set of triads is strongly phase-coupled both at the surface and close to the bed. Meanwhile, the second core of triads is intensified and more organised near the bed due to interactions with the breaking, the undertow, and the bed boundary. The biphase structure shows a more dominant skewed shape at both levels, since it is closer to $0^o$ than to $90^o$. In this position, the model captures additional frequency combinations to reorganise more phases near the bed. Such a condition also indicates that breaking not only adds random turbulence but also enhances triad interactions.

Figure 24a and b present PSD distribution plots past the breaking point. The spectral energy is similar to the breaking point, where a strong, narrow peak is around the carrier. This structure is retained inside the WBL, but with less energy intensity. The self-coupling is high, as indicated by bicoherence values of 0.85-0.87. The sum of the frequencies is larger near the bed than in the free stream, as is the breaking point, indicating a strong influence of the WBL on the triads-coupling. The biphase at this location exceeds the breaking point, with biphase values of 58$^o$ and 31$^o$ at the surface and within the WBL, respectively. The velocity signals are more asymmetrical and less skewed.

Further away from the breaking point, the spectral energy at the *x=145* m position is weak both at the free-stream and near-bed levels. The bicoherence contour along the red line produces $\textrm{b} ^2=$0.70-0.72, indicating strong coupling of the self harmonics. Meanwhile, stronger mixed frequency occurs at the near-bed level, with bed-enhanced extra triads. The biphase demonstrates a sawtooth-like shape at the free-stream and changes to be more skewed at the near-bed.

## Discussion

This study investigates the cross-shore and vertical structure of wave nonlinearity across different breaker types using a coupled framework comprising the phase-resolving Boussinesq model Funwave and a 1DV point-flow model. The Boussinesq component successfully reproduces the overall wave transformation with good fidelity, except in the most energetic stages of plunging breaking, which remain challenging. As a result, the coupled system captures free-stream wave nonlinearity well for both the plunging waves in the SINBAD experiment and the spilling breakers in the LIP11-1C test. The 1DV point flow model, formulated as a first-order boundary-layer representation and consistent with earlier studies^47–49^, provides reliable predictions of near-bed flow structure and nonlinear velocity moments within the wave boundary layer. Its performance supports the applicability of the local hydrostatic assumption beneath progressive surface waves. Overall, the results indicate that a reduced-complexity modelling strategy, e.g, combining accurate phase-resolving wave predictions with simplified vertical flow representation, offers a promising pathway for short- and long-term simulations of sediment transport and beach morphology, provided that surface wave dynamics are well resolved.

Previous studies of wave–boundary-layer processes under oscillatory and weakly breaking waves have shown that boundary-layer filtering enhances near-bed velocity skewness^50^. The present work extends this understanding to strongly breaking conditions on a barred beach, demonstrating that, even in the presence of a plunging jet, splashing bore, strong undertow, and intense turbulence, the increase in *Sk* and decrease in *As* toward the bed persist. This behaviour reflects the relatively weak influence of the undertow close to the bed and the continued presence of a coherent wave boundary layer, despite substantial thinning of the WBL beneath energetic breaking waves. The wave breaking noticeably reduces the magnitude of wave orbital motion throughout the water column. However, the presence of the boundary layer alters the stronger decrease in onshore velocity peak at the free-stream to a larger drop in offshore peak at the near-bed region, as shown in Fig. 9, which results in the decrease in $S_k$ and $A_s$ across the water column in the post-plunging region. These results indicate the importance of resolving WBL for the correct prediction of wave nonlinearity.

The cross-shore evolution of *Sk* and *As* predicted by the model and measured in the experiments shows trends consistent with established concepts of shoaling- and breaking-induced nonlinearity^10,51^. The present study further highlights the important role of breaker type in shaping nonlinear wave statistics. In both test cases, free-stream *Sk* and *As* increase across the shoaling region as wave crests sharpen and troughs broaden, and the phase-shift structure transitions after breaking, from a more substantial up-crossing phase lead to a larger down-crossing phase lead. Bispectral analysis confirms that classical triad interactions elevate the crests.

In the LIP11-1C case (LIP11-1C), both observed and modelled free-stream *Sk* and *As* peak near the breaking point and decay gradually in the surf zone, consistent with field observations under mild conditions^43,44^. In contrast, the plunging SINBAD case exhibits a distinctly non-monotonic response: high skewness and asymmetry in shoaling persist through the initial overturning, then drop sharply at the bar crest where the plunging jet deforms the waveform, and subsequently rise again at the splashing point where the roller enhances crest sharpening. This behaviour departs from the slowly decreasing *Sk* predicted by analytical expressions and underscores the localised nonlinear contributions generated by plunging and splash-driven dynamics. These findings demonstrate that free-stream *Sk* and *As* depend not only on depth and wave height but also strongly on breaker type and the internal structure of the breaking region.

Bispectral analysis provides additional insight into how breaking and boundary-layer processes shape the harmonic content of the velocity signal. Spectral energy decreases with depth in both experiments due to depth-limited attenuation, yet strong triad self-coupling persists, indicating highly organised nonlinearity near the bed. At several locations, mixed-frequency coupling is stronger in the WBL than in the free stream, revealing combined effects of breaking, undertow, and bottom friction. In the plunging SINBAD case, biphase values at the breaking, plunging, and splashing points show a progression from strongly skewed, forward-leaning signals to increasingly sawtooth-like waveforms near the bed, where bicoherence approaches unity. In LIP11-1C, the biphase remains near $0^o$, consistent with skewed but less sawtooth velocity signals maintained by coherent triad coupling at depth. These results reaffirm that shoaling and breaking waves retain strong, organised triad interactions rather than becoming fully random^10,43,52,53^.

Although the present manuscript shows only the hydrodynamics and wave nonlinearity results, the modelling framework also include suspended and near-bed load sediment transport modules. These modules have been validated for both ripple-dominated and sheet-flow conditions (e.g., in our companion studies). However, sediment transport analysis is beyond the scope of this manuscript, which focuses on hydrodynamics and wave nonlinearity.

## Figures and Tables

**Fig. 26:**
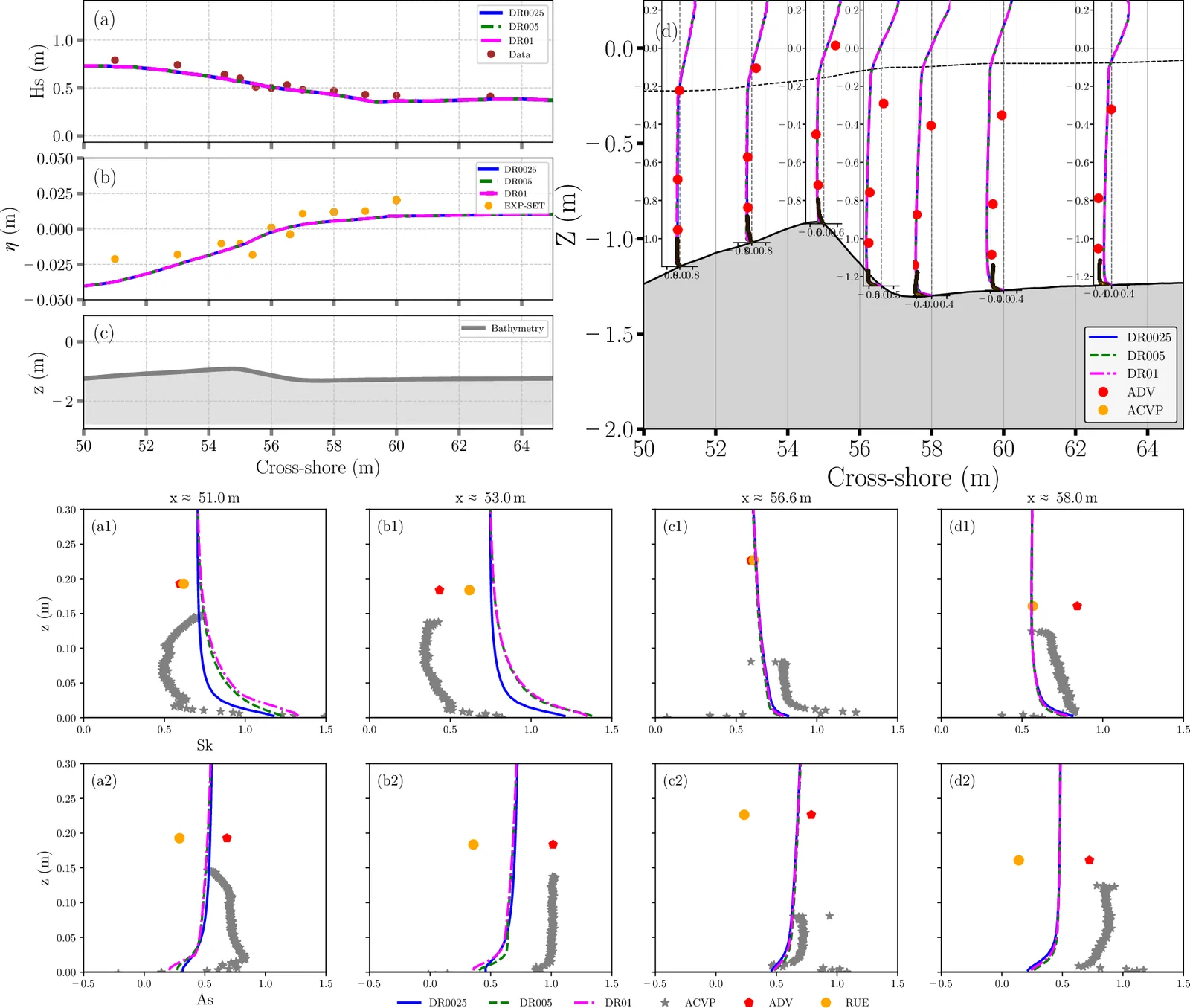
Cross-shore sensitivity of model predictions to the bed-roughness parameter (*DR=0.0025, 0.0005, 0.01*). The panels show the cross-shore distribution of (**a**) wave height, (**b**) mean water level (setup/set down), (**c**) bathymetry, (**d**) undertow, and (a1-d1) velocity skewness and (a2-d2) asymmetry.

**Fig. 27:**
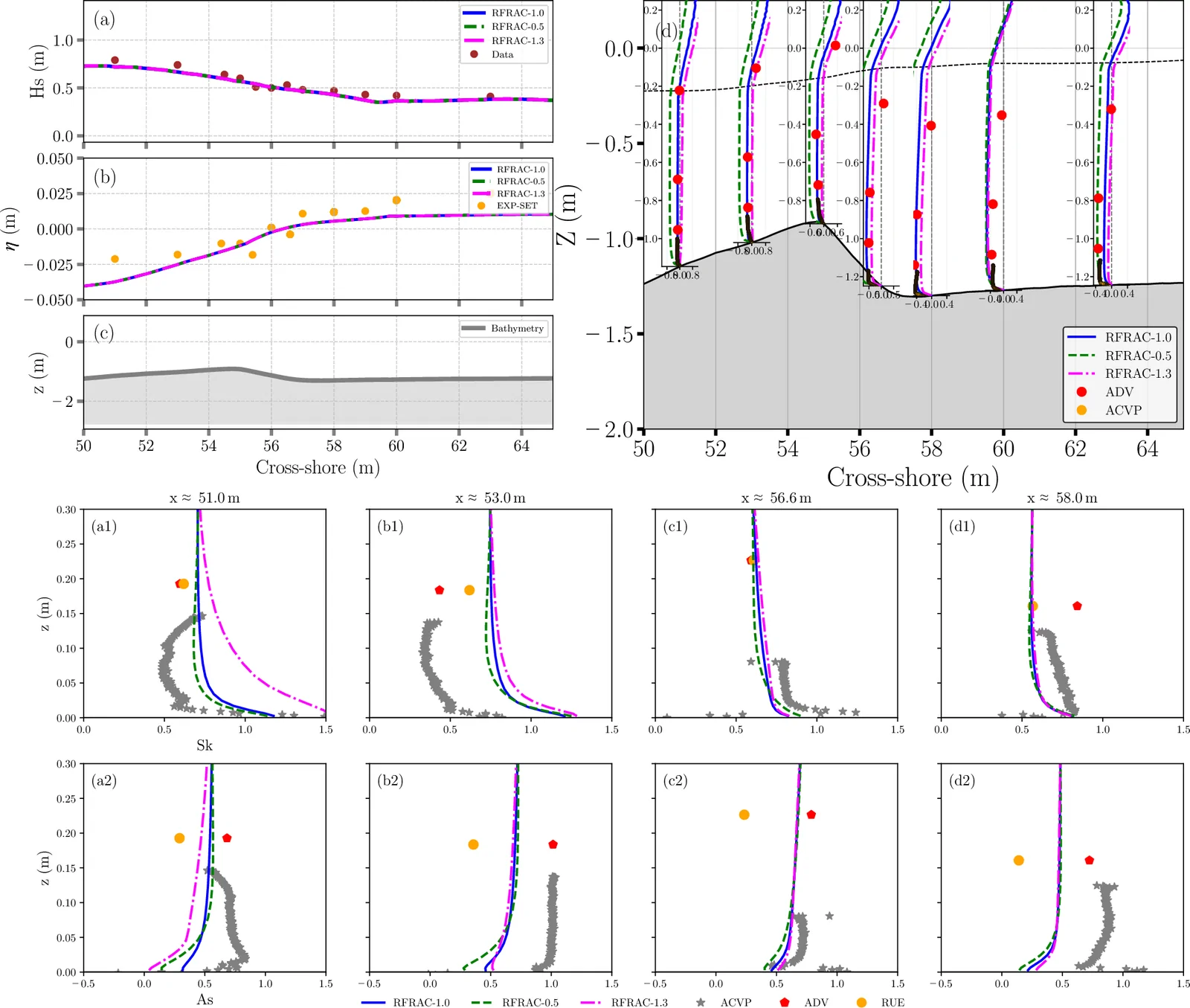
Same as Fig. 26 but for *RFRAC = 0.5, 1.0,1.3*.

**Fig. 28:**
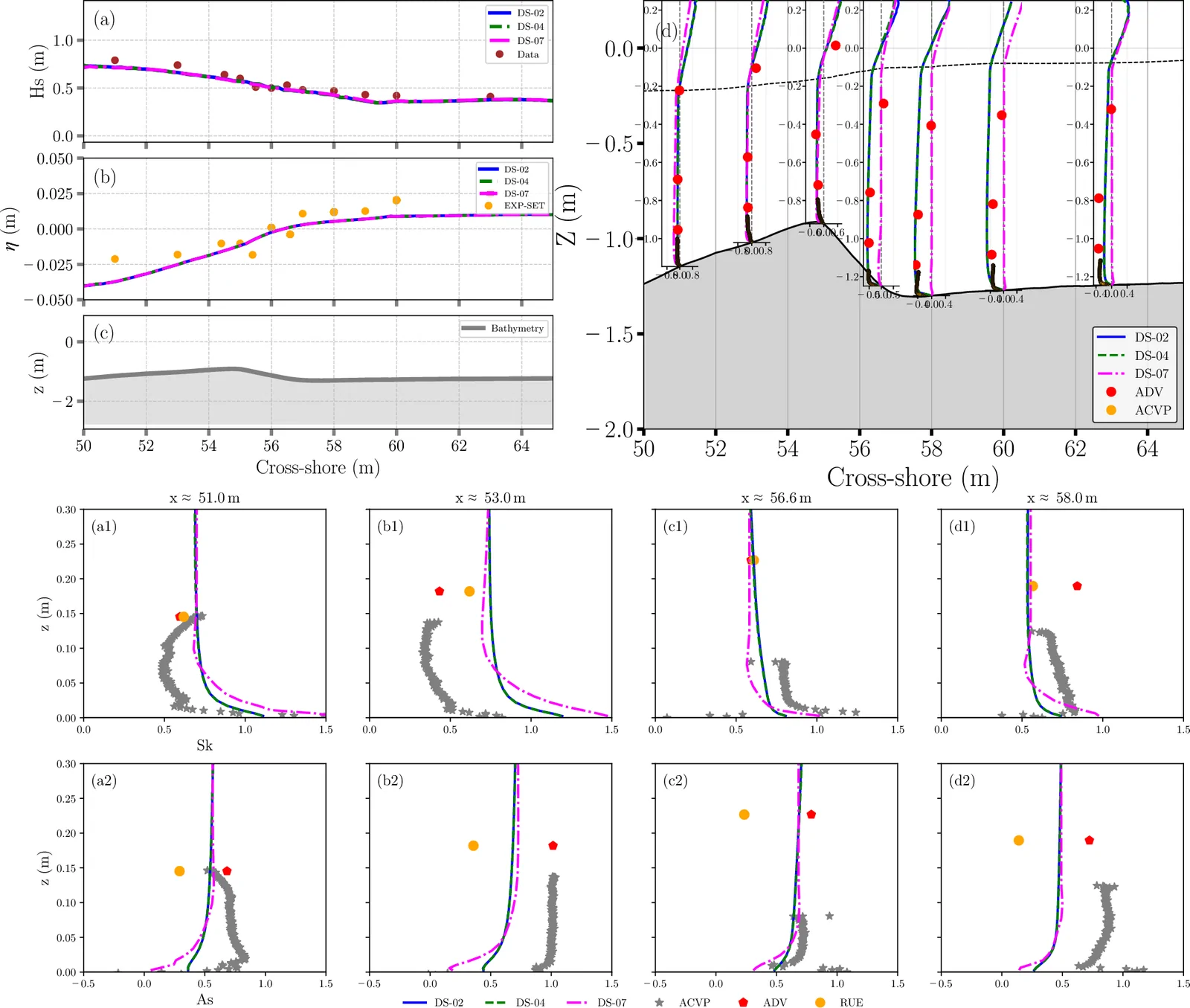
Same as Fig. 26 but for *DS = 0.2, 0.4,0.7*.

## Data Availability

The datasets used for the figures and tables generated by the present study are available from the corresponding author on reasonable request.

## Appendix

### Bispectral analysis methodology

For the bispectral analysis, two approaches were adopted corresponding to the regular-wave (period *T=4* s) and irregular-wave (peak period $T_p=8$ s) experiments. In the regular-wave case, the velocity time series (sampled at *Δ t = 0.1* s) was segmented into individual wave cycles of 40 samples; each segment was multiplied by a Hanning window and zero-padded to an FFT length of 128 points, and ensemble averaging was performed over all cycles (*M ≈ 150*). For the irregular-wave case (sampled at *Δ t = 0.5* s), the time series was divided into segments of 16 samples (one peak period), windowed with a Hanning window, and transformed using an FFT length of 256 points (*M ≈ 100*). In both cases, no overlap was applied because the segmentation was aligned with the dominant wave period. The bispectrum $B(f_1,f_2) = \langle X_m(f_1) X_m(f_2) X_m^*(f_1+f_2) \rangle$ was computed by averaging over all segments, and the squared bicoherence was obtained as

$$b^2(f_1,f_2) = \frac{\left| \sum _m X_m(f_1) X_m(f_2) X_m^*(f_1+f_2) \right| ^2}{\sum _m |X_m(f_1) X_m(f_2)|^2 \; \sum _m |X_m(f_1+f_2)|^2}.$$

Statistical significance was assessed using the fact that under the null hypothesis of no quadratic coupling, $2M b^2$ follows a $\chi ^2_2$ distribution. For a 95% confidence level, the critical bicoherence threshold is $b_{\text {crit}} = \sqrt{\chi ^2_{2,0.95} / (2M)}$; with *M ≈ 150* for the regular waves and *M ≈ 100* for the irregular waves, this yields $b_{\text {crit}} \approx 0.14$ and 0.17, respectively. This methodology ensures reproducibility and allows direct comparison of nonlinear energy transfer across the two wave conditions.

### Sensitivity test

Figs 26, 27, and 28 present the result of model sensitivity test examining the effects of varying roughness, roller coefficient, and breaking slope, respectively. The three sensitivity tests indicate that the wave energy transformation is weakly influenced by the perturbation parameters, as the cross-shore profiles of significant wave height and wave setup/ setdown remain unchanged. In contrast, the undertow shows distinct response to changes in model configuration. Roughness affects only small portion of the shoaling and breaking regions, as shown in Fig. 26a1, b1, suggesting its contribution confined to the near-bed part. The roller coefficient (*RFRAC*) sensitivity test yields minor differences in near-bed skewness and asymmetry (Fig. 27) and has even smaller effects on the wave energy dissipation across the shore. The most notable impact is observed in the undertow distributions, where a higher roller coefficient leads to weaker undertow magnitude in the water column, driven by stronger roller velocity at the surface region. The breaking slope, in contrast, exhibits a substantial influence when different slopes are applied. A steeper breaking slope (*ds=0.7*) shifts the breaking point shoreward, resulting in smaller undertow magnitude around the bar trough (Fig. 28d). This outcome differs from the experimental data. Overall, the sensitivity tests demonstrate model consistency across different configurations.
